# Evaluation of safety and immunogenicity of a group A streptococcus vaccine candidate (MJ8VAX) in a randomized clinical trial

**DOI:** 10.1371/journal.pone.0198658

**Published:** 2018-07-02

**Authors:** Silvana Sekuloski, Michael R. Batzloff, Paul Griffin, William Parsonage, Suzanne Elliott, Jon Hartas, Peter O’Rourke, Louise Marquart, Manisha Pandey, Fran A. Rubin, Jonathan Carapetis, James McCarthy, Michael F. Good

**Affiliations:** 1 Clinical Tropical Medicine Laboratory, QIMR Berghofer Medical Research Institute, Brisbane, Australia; 2 The Institute for Glycomics, Griffith University, Gold Coast, Queensland, Australia; 3 Q-Pharm Pty Ltd, Brisbane, Australia; 4 Department of Medicine and Infectious Diseases, Mater Hospital and Mater Medical Research Institute, Brisbane, Australia; 5 The University of Queensland, Brisbane, Australia; 6 Australian Centre for Health Service Innovation, Queensland University of Technology, Brisbane, Australia; 7 Division of Microbiology and Infectious Diseases, National Institute of Allergy and Infectious Diseases, National Institutes of Health, Bethesda, Maryland, United States of America; 8 Telethon Kids Institute, University of Western Australia and Perth Children’s Hospital, Perth, Australia; University of Auckland, NEW ZEALAND

## Abstract

**Background:**

Group A streptococcus (GAS) is a serious human pathogen that affects people of different ages and socio-economic levels. Although vaccination is potentially one of the most effective methods to control GAS infection and its sequelae, few prototype vaccines have been investigated in humans. In this study, we report the safety and immunogenicity of a novel acetylated peptide-protein conjugate vaccine candidate MJ8VAX (J8-DT), when delivered intramuscularly to healthy adults.

**Methods:**

A randomized, double-blinded, controlled Phase I clinical trial was conducted in 10 healthy adult participants. Participants were randomized 4:1 to receive the vaccine candidate (N = 8) or placebo (N = 2). A single dose of the vaccine candidate (MJ8VAX), contained 50 μg of peptide conjugate (J8-DT) adsorbed onto aluminium hydroxide and re-suspended in PBS in a total volume of 0.5 mL. Safety of the vaccine candidate was assessed by monitoring local and systemic adverse reactions following intramuscular administration. The immunogenicity of the vaccine was assessed by measuring the levels of peptide (anti-J8) and toxoid carrier (anti-DT)—specific antibodies in serum samples.

**Results:**

No serious adverse events were reported over 12 months of study. A total of 13 adverse events (AEs) were recorded, two of which were assessed to be associated with the vaccine. Both were mild in severity. No local reactogenicity was recorded in any of the participants. MJ8VAX was shown to be immunogenic, with increase in vaccine-specific antibodies in the participants who received the vaccine. The maximum level of vaccine-specific antibodies was detected at 28 days post immunization. The level of these antibodies decreased with time during follow-up. Participants who received the vaccine also had a corresponding increase in anti-DT serum antibodies.

**Conclusions:**

Intramuscular administration of MJ8VAX was demonstrated to be safe and immunogenic. The presence of DT in the vaccine formulation resulted in a boost in the level of anti-DT antibodies.

**Trial registration:**

ACTRN12613000030774

## Introduction

*Streptococcus pyogenes*, also known as group A streptococcus (GAS), is a serious human pathogen affecting people of different ages and socio-economic levels. The pathology resulting from GAS infection can be divided into acute suppurative effects and post-streptococcal sequelae. The former includes streptococcal pharyngitis, pyoderma and invasive GAS disease. Post-infectious complications may follow, especially if acute infections are left untreated. These complications include life threatening conditions such as acute rheumatic fever (ARF) leading to rheumatic heart disease (RHD), and acute post-streptococcal glomerulonephritis, which can result in permanent renal damage [1, 2]. Despite advances in diagnosis, and the availability of penicillin for over 70 years, the current global burden of RHD remains high and is associated with increasing prevalence in less developed countries [1, 3–5]. The highest prevalence of RHD in developed countries is in disadvantaged populations who live in remote areas or with limited resources, such as among Indigenous Australians and New Zealanders [1, 6–10]. It has been estimated that there are 33,438,800 prevalent cases of RHD globally, with 10,513,200 disability-adjusted life years lost annually from RHD, and around 319,400 deaths due to RHD each year [5]. Additionally, invasive GAS disease is estimated to be responsible for a further 160,000 deaths per year [11]. Limitations for an accurate quantification of the global burden and mortality caused by GAS globally have been reported; thus, it is very likely that these numbers are much higher than indicated [7, 12].

Presently, there is no strategy for prevention of GAS infection or its sequelae, thus highlighting the need for a prophylactic vaccine. Vaccine research has mainly focused on the M-protein, a major virulence factor found on the surface of the bacteria. The highly variable amino acid sequence at the N terminus of the M-protein determines the serotype of the M-protein [13, 14]. This part of the protein has also been found to be highly immunogenic and responsible for eliciting strain-specific immunity to GAS. However, a challenge to the identification and development of a M protein-specific vaccine is the potential for epitopes of the M protein to elicit cross-reactive immune responses to human tissues and/or promote proliferation of cross-reactive T-cells that in turn may contribute to the pathology of RHD [15–18]. Different approaches have been utilized to overcome these limitations and to develop a vaccine that could elicit a protective immune response without the risk of inducing host tissue cross–reacting antibodies. Significant progress has been made in the development of a recombinant multi-valent vaccine that consists of multiple amino-terminal epitopes of the M-protein that are prevalent mainly in North America and Europe [19–21]. These studies have demonstrated that this approach can result in the induction of an antibody response capable of opsonizing the specific strains of the M-type protein represented in the vaccine [20]as well as the closely related sub M-type variants [22]. Nevertheless, the specificity of the induced *emm*–type antibodies, limits the use of this approach to GAS vaccine development, especially in developing countries where the prevalence of GAS infections is very high with a rapid turnover of M-type variants, resulting in occurrence of GAS strains that differ significantly from those found in North America and Europe [23, 24].

The use of conserved GAS epitopes in vaccine development has the advantage of inducing protection against infections caused by different GAS strains worldwide including in both developed and less developed countries [25, 26]. In this respect, the carboxyl terminus of the M-protein is highly conserved across different GAS strains [14, 27, 28] and therefore represents an attractive target.

A novel conjugate vaccine candidate that consists of a synthesized and acetylated peptide antigen (J8) from the conserved carboxyl terminus region of the M-protein has been described [29]. This peptide was conjugated to Diphtheria Toxoid (DT) as a carrier protein, and formulated with alum (Alhydrogel, 2% Aluminium hydroxide) adjuvant. In murine models of infection this vaccine was able to prevent invasive and skin disease [26, 30, 31].

Pre-clinical studies in rabbits demonstrated that repeated administration of J8-DT with Alum by IM injection was well tolerated and there were no toxicologically significant findings noted [32]. Furthermore, this vaccine formulation did not induce the immunopathogenesis of RF/RHD when administered with or without Freund’s Adjuvant in rat RF/RHD model studies [32] in which autoimmune valvulitis can be readily induced following immunization with full length M protein in complete Freund’s Adjuvant and *Bordetella pertussis* [33].

The aim of the present study was to evaluate the safety and immunogenicity of this novel vaccine candidate MJ8VAX, when delivered intramuscularly to healthy adults. The placebo participants received normal saline.

## Materials and methods

The protocol, the echocardiographic exclusion criteria, the ECHO results and supporting CONSORT checklist are available as supporting information: see S1 Protocol, S1 Echocardiographic exclusion criteria and S1 CONSORT Checklist.

### Ethics statement

This study was approved by the QIMR Berghofer Human Research Ethics Committee. The study was conducted in accordance with the principles of the Declaration of Helsinki (Recommendations guiding Medical Doctors in Biomedical Research Involving Human Participants, 1964 and subsequent updates), and with the NHMRC National Statement on Ethical Conduct in Human Research (2007). The study was conducted under a CTN scheme and it was registered on the Australian New Zealand Clinical Trials Registry as required by the WHO and ICMJE, with the trial reference ACTRN12613000030774. All participants gave written informed consent before being included in the study.

### Vaccine candidate

The antigen component in the proposed vaccine candidate was a 29 amino acids long peptide epitope (QAEDKVKQSREAKKQVEKALKQLEDKVQ) copying the sequence from the C terminus of the M protein conjugated to carrier molecule as previously reported [32]. Following the synthesis on a solid phase resin, the J8 peptide was acetylated, purified using preparative HPLC and then conjugated to diphtheria toxoid (DT) as a ‘carrier’ molecule. To facilitate the conjugation to the diphtheria toxoid carrier molecule using 6’-maleimido-caproyl n-hydroxy succinimide (MCS) linker [34], the peptide was synthesized to contain a cysteine (Cys) residue on the C-terminus. Amino acid analysis has shown that the substitution ratio between the peptide and the carrier molecule ranged between 8 to 14 molecules of J8 peptides to 1 molecule of DT and the net w/w was between 310–440 μg of J8 peptide and 560–690μg of DT per mg of J8-DT conjugate. The J8-DT conjugate was then adsorbed onto alum (2% Aluminum hydroxide, Alhydrogel). The final vaccine formulation also contained phosphate as a pH stabilizer in an isotonic 0.9% sodium chloride solution. Each 0.5 mL dose of the vaccine candidate (MJ8VAX) contained approximately 15 μg of J8 peptide, 35 μg of diphtheria toxoid and between 315–715 μg elemental aluminum as determined by assay. The vaccine formulation was a sterile, white, homogeneous suspension intended for intramuscular injection that was supplied in single dose vials. The placebo injection contained 0.5 mL of a clinical grade, ready-to-use sterile 0.9% sodium chloride (normal saline) solution.

### Study design and procedures

This study was a randomized, double-blinded, controlled Phase I clinical trial conducted at the contract research organization Q-Pharm Pty Ltd (Queensland, Australia). The participants were to be randomized to receive the vaccine candidate (N = 15) or saline as placebo control (N = 5). The randomization was to be conducted in 2 blocks, the first with 2 sentinels (1 active and 1 placebo) on Day 1 and the second for the remaining 18 (14 active and 4 placebo) subsequently. The randomization was computer generated by selecting a random permutation of the appropriate number of actives and placebos in each block. The statistical package “R” was used for randomization.

Participants were allocated a randomization number on enrolment and all study personnel including the investigators, nurses and laboratory personnel were blinded to the randomization scheme with exception to the authorized study pharmacist involved only in preparation and labelling of the doses for administration. A code list for emergency unblinding purposes was kept in a secure place at the clinical site by the study pharmacist.

Initially the study was planned to include administration of two doses of MJ8VAX vaccine candidate. However, following the administration of the first dose, the study was placed on hold at the sponsor’s request. Approval was subsequently gained to continue the study. Due to the prolonged schedule for administration of the second vaccine dose, the sponsor in consultation with the Investigator, recommended no additional doses be administered and the study to continue with assessment of safety and interim immunogenicity of the single vaccine dose.

### Participants

Participants enrolled in the study were non-smokers, healthy male and female adult volunteers, with BMI in the range of 18 to 30 kg/m^2^ and who met all of the inclusion and none of the exclusion criteria. Women participants were required not to become pregnant for a minimum of 180 days following the vaccination in the study. Exclusion criteria also included evidence for recent GAS infection as measured by anti-streptolysin O or anti-DNase B levels, positive GAS throat culture at the screening visit or positive rapid antigen test result on the day of dosing, pre-existing anti-J8 antibodies levels and receipt of any diphtheria toxoid-containing vaccine within the previous 5 years (such as DTP, Hepatitis A, Boostrix, Adacel or Menactra). Details of the inclusion and exclusion criteria are presented in study protocol (S1 Protocol).

### Assessment of safety

Two of the enrolled participants were initially dosed as sentinels, one randomized to receive placebo and the other the vaccine candidate. These participants were observed for at least 6 hours for any immediate adverse events. The Investigator and the Independent Safety Monitor reviewed the data and ensured that no immediate AEs had occurred before the remaining enrolled participants were dosed. The remaining participants were dosed at least 24 hours following the initial dosing of the first two participants. Each dose was administered as an intramuscular injection of 0.5 mL volume. Following the dosing, the participants were monitored for at least 30 minutes for evaluation of any immediate adverse reactions. Throat swabs were taken from each participant at screening and on the day of vaccine administration and a rapid antigen test for the presence of GAS was performed prior to vaccine administration. A follow up phone call was made to all participants by the clinical personnel on Day 2 and Day 8 following the dosing to ensure that the participants had not experienced any significant AE. The participants were asked to record in a memory aid and report on any vaccine reactions or AEs that they might experience up to 7-days post immunization. AE and serious adverse event (SAE) reporting and assessment was conducted throughout the study at the scheduled clinic visits. AEs were monitored via telephone, within the clinical research unit, and on an outpatient basis after dosing. Unsolicited and solicited AE were reported throughout the duration of the study. All of the recorded AEs were tabulated and graded for severity (mild, moderate and severe) and relationship (associated and non-associated) to vaccine administration. All participants were encouraged to contact the clinical investigators or to visit the clinical site if they experienced any severe AE reactions following each immunization, or if they had any concern.

Biochemical and hematologic analyses for safety parameters including Anti-streptolysin O (ASOT) and α-DNase B tests and urinalysis were performed for all participants at screening, prior to the injection and then at Day 28 after the administered dose and at follow-up visits on Days 180, 266 and 350. A urine pregnancy test was also performed for all female participants on the day of vaccination. Serum samples collected from each participant at time points prior to the injection, and at the final study visit on Day 350, are currently kept for use in testing for the presence of human tissue cross-reactive antibodies should suitable assays for detection of cross reactive become available.

### Echocardiography

Transthoracic echocardiography (ECHO) and electrocardiogram (ECG) tests were performed for each participant in this study at screening, 28 days after vaccination and at the final visit on day 350 or on the day of early termination by a technician blinded to the type of treatment that the participants were receiving. All echocardiography was performed using the same ultrasound equipment (General Electric Healthcare Vivid 7) by a single echocardiographer. A single cardiologist (WP) interpreted all echocardiograms using a pre-specified protocol in a blinded fashion. The protocol specified that if any findings were identified as abnormal a second cardiologist was to be asked to independently review the test results and report on the findings. Stringent exclusion criteria were applied at baseline to exclude variations of normal echocardiography that might adversely affect reproducibility at follow up. Details of the echocardiographic exclusion criteria are presented in S1 Echocardiographic exclusion criteria. Echocardiographic endpoints for follow up scans were also biased towards sensitivity in detecting any change in order to emphasise safety of the protocol (see S1 Table for more details).

### Assessment of vaccine immunogenicity

Immunogenicity of the vaccine was determined by measuring the serum level of specific antibodies to the J8 peptide-specific and diphtheria toxoid on the day of vaccination (Day 0), 28 days after the vaccination and at follow-up visits on Days 180, 266 and 350. Standard ELISA assays were performed with three technical replicates to determine the level of both J8 peptide-specific and DT specific serum IgG concentrations (μg/mL) in sera of all participants. Changes in levels of anti-J8 antibodies were monitored to assess the kinetics of the antibody response after vaccination. Purified human J8 peptide-specific antibodies from sera obtained from donors were used to generate a standard curve, as well as a positive control for each ELISA plate as previously described [30]. The level of anti-DT antibodies in each participant was measured prior to the injection and at the end of the study. The significance of the levels of anti-J8 and anti-DT antibodies measured following the vaccination was determined using one-tailed Mann-Whitney test, not corrected, (PRISM Version 5.0C).

### Sample size and statistical analysis

This study was a Phase I safety and immunogenicity single cohort clinical trial planned to consist of N = 15 investigational vaccine recipients and N = 5 control saline recipients. Saline recipients were included to blind clinical and laboratory assessments. They also served as the basis of comparison should any study product safety concerns to be detected. The sample size chosen was not anticipated to provide sufficient power to test for statistically significant differences or similarities amongst the study participants; thus, no formal statistical analyses were planned and preference was given to reporting summary measures rather than formal hypothesis testing. All analyses were regarded as exploratory, and any significant findings were regarded as hypothesis-generating, rather than hypothesis-confirming.

## Results

### Participants

It was planned to recruit 22 participants in order to enrol 20 participants into the study. Thirty-eight volunteers were screened and ten participants were enrolled in the study and dosed. Twenty-eight of the screened volunteers failed to meet the inclusion/exclusion criteria. Causes of screening failure included out-of-range pathology results, out-of-range pre-existing anti-J8 antibodies, positive ASOT or anti-DNAse B, evidence of current GAS infection, urine drug positivity and/or unsuitable baseline echocardiograms.

**Fig 1:**
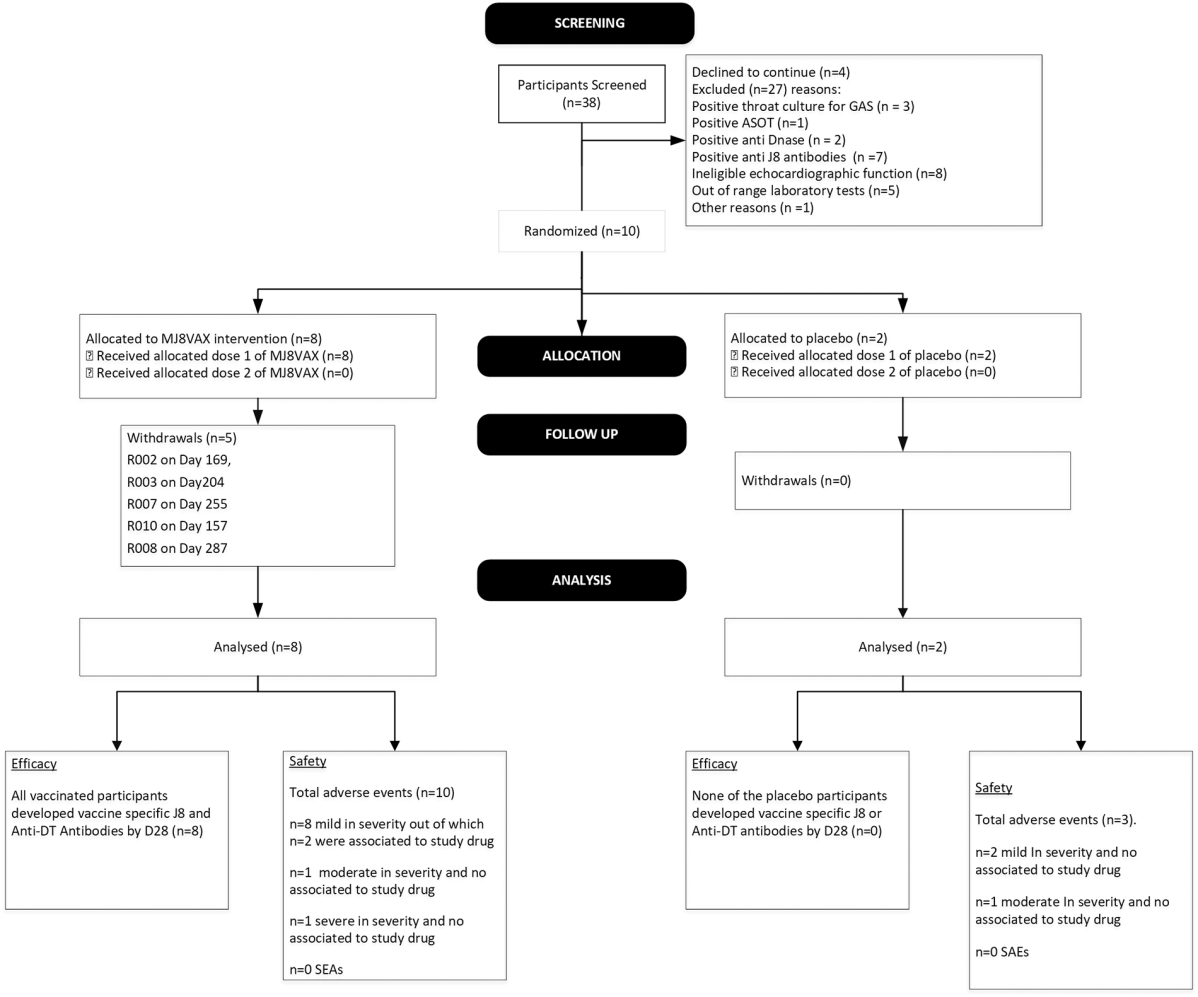
Participation flow. Ten (10) out of 38 participants that were screened for this study, were randomized to receive either the vaccine candidate or placebo. None of the participants received a second vaccination. Five of participants that received a vaccine candidate withdrew from the study by Day 287. All efficacy and safety data recorded at different time points for the participants in this study were included in the analysis.

Fig 1 illustrates the flow of participants from screening until the completion of the study. Due to an extension of time required to recruit all twenty healthy volunteers and the risk to prolong the overall duration of this study, a decision was made to proceed with the study following enrolment of the ten participants. The age range of the enrolled participants was 20–44 (mean 31 years, Table 1). Four of the enrolled ten participants were female and six were male. Nine of the enrolled participants were Caucasian and one was an Indigenous Australian. A total of five participants who received the study product completed the study on Day 350. At the Sponsors’ request, the study was placed on a temporary hold after the first dose of vaccine/placebo had been administered because of a contractual issue that arose with a supplier of a component of the vaccine. This was not considered to be a safety concern for the participants or for the integrity of the vaccine and its components. The study hold however, adversely affected the study schedule and the volunteers’ willingness to continue their participation in the study. For this reason the Investigator in consultation with the Sponsor decided to continue the study without administration of the second dose. Five of the enrolled participants who had received the study product withdrew from the study between 6 to 10 months following the vaccination. There were no withdrawals during the study attributed to the study vaccine.

**Table 1:** Demographic profile of enrolled participants.

Age (years)	Mean ± SD	30.3 ± 8.7
	Range	20–44
Age Groups	18–29	6 (60%)
	30–39	2 (20%)
	40–45	2 (20%)
Sex	Male	6 (60%)
	Female	4 (40%)
Race	Caucasian	9 (90%)
	Australian Aborigine/Torres Strait Islander	1 (10%)
BMI	Mean ± SD	23.4
	Range	18.5–27.9
Height (cm)	Mean ± SD	173.4
	Range	162–187
Weight (kg)	Mean ± SD	70.6
	Range	49.8–85.5

### Participant flow

Eight (8) of the ten (10) participants were randomized to receive MJ8VAX vaccine dose and two of the participants to received placebo (control) (Fig 1). The extension of the study duration caused five (5) of the enrolled participants to withdraw from the study and the remaining completed the study on Day 350.

### Safety assessment

Thirteen AEs were observed in the course of this study; two of these were assessed as being associated to the investigational vaccine (Table 2). One of these two AEs (headache), resolved following treatment with paracetamol; the other AE, abdominal discomfort (attributable to irritable bowel syndrome) resolved with high fibre supplements. Both AEs were mild in severity. Two out of the thirteen AEs that were recorded during the study were considered as moderate in severity and one was severe; none were associated with the investigational vaccine. Three of the recorded AEs had not resolved at the end of the study, (scabies, bulging disc and back pain). No SAEs were reported in any of the immunized participants during the course of the study. No clinically significant changes in clinical cardiac examinations were recorded, apart from a non-clinical significant sinus bradycardia recorded on Day 180 in one participant by ECG. No other changes in echocardiographic examinations or ECG findings were identified. No clinically significant changes in laboratory results were observed; specifically no changes in antistreptococcal antibodies (ASOT and anti-DNase B) were observed.

**Table 2:** AEs recorded in the study.

Randomisation Number	Event	Duration	Severity	Relationship to Study Vaccine	Actions/Comments
R001	Scabies	Ongoing	Moderate	Not associated	Treated with promethazine hydrochloride and permethrin.
R003	Urinary tract infection	<5 days	Mild	Not associated	Resolved following treatment with trimethoprim and codeine phosphate and paracetamol.
	Headache	3 hours	Mild	Not associated	Resolved following treatment with paracetamol and pseudoephedrine hydrochloride, paracetamol and codeine phosphate.
	Migraine	9 hours	Mild	Not associated	Resolved following treatment with codeine phosphate and paracetamol.
	Bulging Disc	Ongoing	Severe	Not associated	Resolved following treatment with diazepam, codeine phosphate and paracetamol, oxycodone hydrochloride, paracetamol and ibuprofen.
R004	Symptoms of irritable bowel	< 10 months	Mild	Associated	Resolved following treatment with high fibre supplements.
	Generalised body rash	<11 months	Mild	Not associated	Resolved following treatment with fexofenadine.
	Headache	3 hours	Mild	Not associated	Resolved following treatment with paracetamol.
R005	Abdominal pain	60 minutes	Moderate	Not associated	Resolved without treatment.
R008	Headache	12 hours	Mild	Associated	Resolved following treatment with paracetamol.
R009	Back pain	Ongoing	Mild	Not associated	Treated with ibuprofen.
	Skin lesions	<14 days	Mild	Not associated	Resolved following excision performed under local anaesthetic.
R010	Upper respiratory tract infection	5 days	Mild	Not associated	Resolved following treatment with ibuprofen.

### Analysis of vaccine immunogenicity

**Fig 2:**
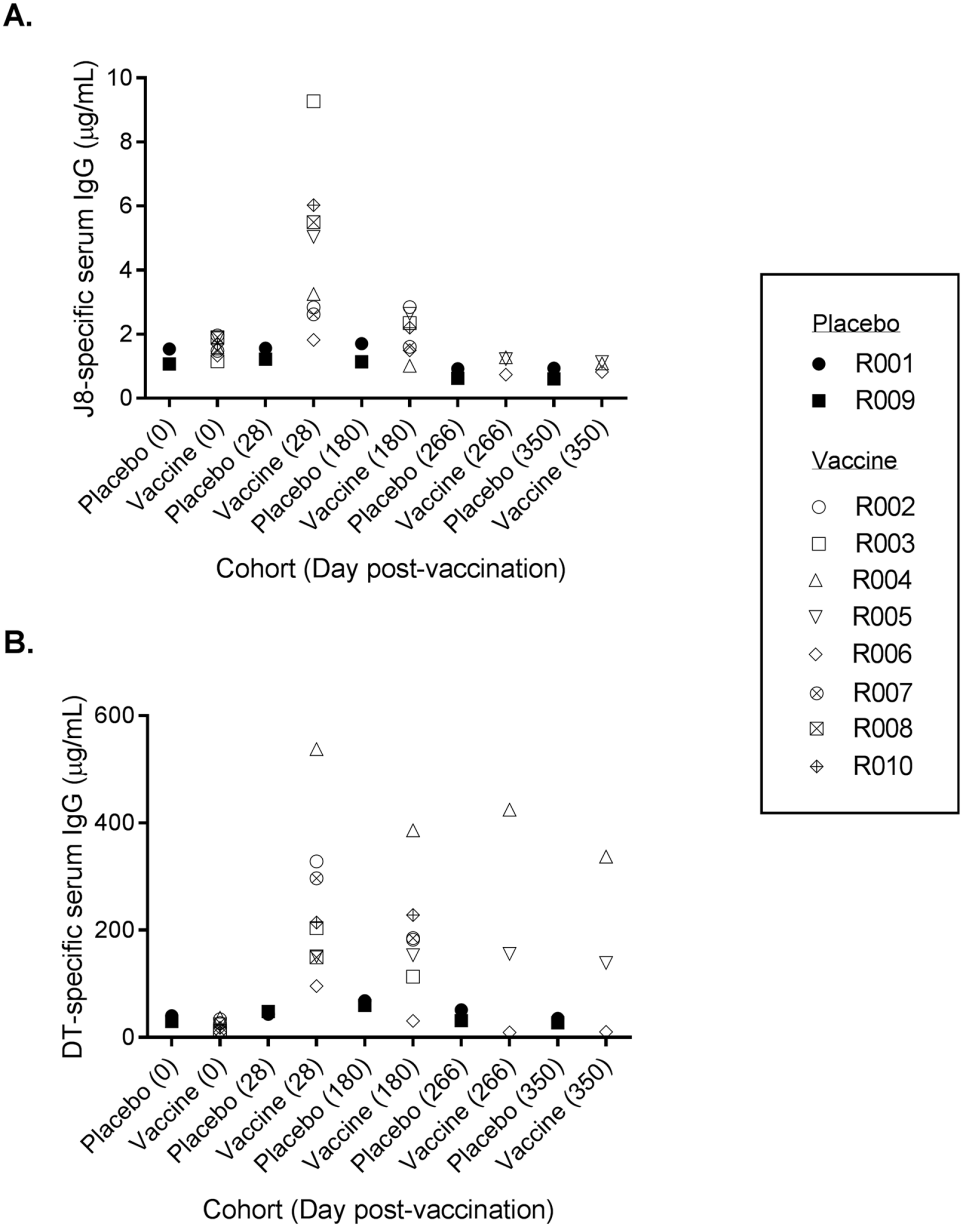
J8-specific (Panel A) and DT-specific (Panel B) serum IgG concentrations for each participant at Days 0, 28, 180, 266 and 350.

The concentration of J8 peptide-specific serum IgG and anti-DT serum IgG for each participant at Days 0, 28, 180, 266 and 350 is illustrated in Fig 2. The raw data is included in S2 Table. On Day 0, the concentration of anti-J8 IgG ranged from 1.07–1.96 μg/mL for all participants. Although there was considerable inter-subject variability in the antibody response on Day 28, there was a clear trend of increased anti-J8 IgG antibody levels among participants who were administered the MJ8VAX dose (1.82–9.27 μg/mL). In comparison, there was no increase in anti-J8 IgG observed in the 2 subjects administered the placebo (1.22–1.56 μg/mL). On Day 180, anti-J8 IgG concentrations in the vaccinated cohort decreased to levels close to baseline (1.01–2.84 μg/mL) and remained at baseline levels on Day 266 and Day 350.

The concentration of anti-DT IgG on Day 0 ranged from 9.29–40.33 μg/mL for all participants. Similar to the results for anti-J8 IgG, there was considerable inter-subject variability in the antibody response on Day 28 but there was a clear trend of increased anti-DT IgG concentrations in the cohort of participants who were administered the MJ8VAX dose (95.62–537.99 μg/mL). Anti-DT IgG concentrations in the 2 participants administered the placebo remained close to baseline levels (43.08–48.21 μg/mL). However, in contrast to the results for anti-J8 IgG, anti-DT IgG concentrations remained elevated in most participants in the vaccinated cohort on Day 180 (30.56–386.39 μg/mL) and in 2 of the 3 participants who were analysed in the vaccinated cohort on Days 266 and 350.

## Discussion

This is the first study to evaluate the safety and immunogenicity of the vaccine (MJ8VAX) containing a peptide from the conserved region of the GAS M protein. It demonstrated that a single intramuscular dose of the vaccine was safe, well tolerated and immunogenic, with participants who received the active vaccine developing specific antibody responses to J8 and DT by Day 28 following the immunization. A vaccine-specific antibody response was detectable in the active participants on Day 180 following the vaccination, but their levels were lower than on Day 28. Although similar findings were observed for anti-DT antibodies in the active participants, the level of anti-DT antibodies detected on Day 180 remained higher, possibly due to previous vaccination in childhood.

To achieve a long term protection, a multiple-dose regime would most likely be required. This study was designed to investigate a two dose regime of vaccination, but with the study hold that delayed the time window for administration of the second vaccine dose, firm conclusions about the safety and immunogenicity of this vaccine are not possible. Additional limitation of this study is that only 10 participants of the planned 20 were enrolled, reducing data available for analysis.

Measurement of the immunogenicity allowed us to demonstrate that a single dose of J8-DT vaccine formulation could elicit vaccine specific immune response, however the overall immunogenicity was considered to be low. Previous studies in mice have shown that multiple doses of J8-DT are required to protect against GAS skin disease and therefore, the bactericidal activity of the antisera in this study following a single dose vaccination was not investigated.

Further clinical investigations will be required to assess the number of doses that would elicit an optimal level and duration of protection against GAS infection. Irrespective of the limitations of the immunogenic effect of the tested vaccine, the findings in this study provide a basis for further investigation in determining a successful vaccination schedule that could provide long term protection across different strains of GAS, regardless of their geographical prevalence.

The results indicated that incorporation of the conserved region of GAS in the vaccine formulation offers an alternative approach for design of an anti-GAS vaccine. Incorporating a conserved region in a vaccine formulation could result in the added benefit of providing protection against GAS infections globally. A vaccine formulation effective across different GAS strains may in turn contribute to reducing the risk of secondary and close contact cases of invasive GAS diseases [35, 36].

Further investigations and changes in the formulation of the vaccine candidate are also required to evaluate the possibility for enhancing the immunogenicity of the vaccine candidate. Studies in mice and non-human primates have shown that replacing DT as a carrier protein with CRM197 to create J8-CRM197 does not affect the overall immunogenicity of the J8 peptide [37]. Different modes for anti GAS vaccine delivery such as intranasal vaccination using multicomponent lipid core peptides (LCP) construct have also been investigated [38, 39].

In summary, this is a first report that shows that the conserved region of the GAS M protein can be used to design a safe vaccine candidate that could be effective against different strains of GAS. Further investigations for the purpose of improving the dosing and enhancing the immunogenicity of the vaccine would be required to determine the optimal number of vaccinations to elicit a long term protective immune response.

## Supporting information

S1 Protocol A randomized, double blinded within dose, controlled, safety and immunogenicity study of group a streptococcus vaccine candidate in healthy participants. (DOC)

S1 Echocardiographic Exclusion Criteria (DOCX)

S1 CONSORT Checklist (DOC)

S1 Table ECHO results. (DOCX)

S2 Table J8 IgG and DT IgG serum concentration (μg/mL). (DOCX)
