# Supplementary material for: Evaluation of safety and immunogenicity of a group A streptococcus vaccine candidate (MJ8VAX) in a randomized clinical trial
Source: PLoS One. 2018 Jul 2;13(7):e0198658. doi: 10.1371/journal.pone.0198658 (PMC6028081; doi:10.1371/journal.pone.0198658)
Supplement: S1 Protocol — (DOC) [file pone.0198658.s001.doc]

**S1 Protocol. A Randomized, Double Blinded Within Dose, Controlled, Safety and Immunogenicity Study of Group A Streptococcus Vaccine Candidate in Healthy Participants**

**Sponsor for Therapeutic Goods Administration (Australia):**

*Queensland Institute of Medical Research (Australia)*

**Program Investigator:** Dr. Michael F. Good, B.Sc. (Med), MBBS, Ph.D., M.D., D.Sc

**Clinical Investigator:** Dr James S. McCarthy, M.D., FRACP

**Draft or Version Number:**

3.0

**Day Month Year**
8 March *2013*

**Investigator Signature Page**

I have read the protocol and agree that it contains all necessary details for carrying out the study as described. I will conduct this protocol as outlined herein and will make a reasonable effort to complete the study within the time designated.

I agree to personally conduct or supervise the described Study.

The study will be conducted in accordance with the following:

- World Medical Association Declaration of Helsinki – Ethical Principles for Medical Research Involving Human Subjects
- NH&MRC National Statement on Ethical Conduct in Human Research Humans (2007).
- Notes for Guidance on Good Clinical Practice – Annotated with TGA Comments (CPMP/ICH/135/95), as adopted by the Australian Therapeutic Goods Administration (July 2000).
- Current ethics approved Clinical Trial Protocol

I agree to inform all Participants that the study drug is being used for investigational purposes and I will ensure that the requirements related to obtaining informed consent are in accordance with ICH Guidelines for Good Clinical Practices (GCP) section 4.8 and local requirements.

I agree to report adverse events that occur in the course of the Study to the sponsor in accordance with ICH Guidelines for Good Clinical Practices (GCP) section 4.11 and local requirements.

I have read and understand the information in the Investigator’s Brochure, including the potential risks and side effects of the study drug.

I agree to promptly report to the Ethics Committee (EC) all changes in the research activity and all unanticipated problems involving risk to Participants. I will not make any changes to the conduct of the study without EC and Sponsor approval, except when necessary to eliminate apparent immediate harm to Participants.

I agree to maintain adequate and accurate records and make those records available in accordance with ICH Guidelines for Good Clinical Practices (GCP) section 4.11 and local requirements.

I agree to ensure that all associates, colleagues, and employees assisting in the conduct of the study are informed about their obligations in meeting the above commitments.

I understand that the Study may be terminated or enrollment suspended at any time by the sponsor, with or without cause, or by me if it becomes necessary to protect the best interest of the Participants.

_______________________________________ Date: _____________________________

James S McCarthy MBBS

Principal Investigator

Table of Contents

Page

[Table of Contents iii](#__RefHeading___Toc339612592)

[List of Abbreviations vi](#__RefHeading___Toc339612593)

[Protocol Summary viii](#__RefHeading___Toc339612594)

[1 Key Roles 1](#__RefHeading___Toc339612595)

[2 Background Information and Scientific Rationale 5](#__RefHeading___Toc339612596)

[2.1 Background Information 5](#__RefHeading___Toc339612597)

[2.2 Rationale 9](#__RefHeading___Toc339612598)

[2.3 Potential Risks and Benefits 11](#__RefHeading___Toc339612599)

[2.3.1 Potential Risks 11](#__RefHeading___Toc339612600)

[2.3.2 Known Potential Benefits 11](#__RefHeading___Toc339612601)

[3 Objectives 12](#__RefHeading___Toc339612602)

[3.1 Study Objectives 12](#__RefHeading___Toc339612603)

[3.2 Study Outcome Measures 12](#__RefHeading___Toc339612604)

[3.2.1 Primary Outcome Measures 12](#__RefHeading___Toc339612605)

[3.2.2 Secondary Outcome Measures 12](#__RefHeading___Toc339612606)

[4 Study Design 13](#__RefHeading___Toc339612607)

[5 Study Enrollment and Withdrawal 15](#__RefHeading___Toc339612608)

[5.1 Participant Inclusion Criteria 15](#__RefHeading___Toc339612609)

[5.2 Participant Exclusion Criteria 16](#__RefHeading___Toc339612610)

[5.3 Treatment Assignment Procedures 19](#__RefHeading___Toc339612611)

[5.3.1 Randomization Procedures 19](#__RefHeading___Toc339612612)

[5.3.2 Masking Procedures 19](#__RefHeading___Toc339612613)

[5.3.3 Termination of Study 20](#__RefHeading___Toc339612614)

[5.3.4 Reasons for Withdrawal 20](#__RefHeading___Toc339612615)

[5.3.5 Handling of Withdrawals 20](#__RefHeading___Toc339612616)

[6 Study Intervention/Investigational Product 21](#__RefHeading___Toc339612617)

[6.1 Study Product Description 21](#__RefHeading___Toc339612618)

[6.1.1 Acquisition 21](#__RefHeading___Toc339612619)

[6.1.2 Formulation, Packaging, and Labeling 21](#__RefHeading___Toc339612620)

[6.1.3 Product Storage and Stability 21](#__RefHeading___Toc339612621)

[6.2 Dosage, Preparation and Administration of Study Intervention/Investigational Product 22](#__RefHeading___Toc339612622)

[6.3 Accountability Procedures 23](#__RefHeading___Toc339612623)

[6.4 Concomitant Medications/Treatments 23](#__RefHeading___Toc339612624)

[7 Study Schedule 25](#__RefHeading___Toc339612625)

[7.1 Screening Period (Day –28 to Day –7) 25](#__RefHeading___Toc339612626)

[7.2 Enrollment/Baseline 26](#__RefHeading___Toc339612627)

[7.3 Treatment Period 27](#__RefHeading___Toc339612628)

[7.4 Follow-up 30](#__RefHeading___Toc339612629)

[7.5 Final Study Visit 31](#__RefHeading___Toc339612630)

[7.6 Early Termination Visit 32](#__RefHeading___Toc339612631)

[7.7 Unscheduled Visit 33](#__RefHeading___Toc339612632)

[8 Study Procedures/Evaluations 34](#__RefHeading___Toc339612633)

[8.1 Clinical Evaluations 34](#__RefHeading___Toc339612634)

[8.2 Laboratory Evaluations 35](#__RefHeading___Toc339612635)

[8.2.1 Clinical Laboratory Evaluations 35](#__RefHeading___Toc339612636)

[8.2.2 Special Assays or Procedures 37](#__RefHeading___Toc339612637)

[8.2.3 Specimen Preparation, Handling, and Shipping 37](#__RefHeading___Toc339612638)

[9 Assessment of Safety 39](#__RefHeading___Toc339612642)

[9.1 Specification of Safety Parameters 39](#__RefHeading___Toc339612643)

[9.2 Methods and Timing for Assessing, Recording, and Analyzing Safety Parameters 39](#__RefHeading___Toc339612644)

[9.2.1 Adverse Events 39](#__RefHeading___Toc339612645)

[9.2.2 Serious Adverse Events 43](#__RefHeading___Toc339612646)

[9.2.3 Procedures to be Followed in the Event of Abnormal Laboratory Test Values or Abnormal Clinical Findings 43](#__RefHeading___Toc339612647)

[9.3 Reporting Procedures 44](#__RefHeading___Toc339612649)

[9.3.1 Serious Adverse Events 44](#__RefHeading___Toc339612650)

[9.3.2 Regulatory Requirements 45](#__RefHeading___Toc339612651)

[9.3.3 Reporting of Pregnancy 46](#__RefHeading___Toc339612652)

[9.4 Type and Duration of Follow-up of Subjects after Adverse Events 46](#__RefHeading___Toc339612653)

[9.5 Halting Rules 46](#__RefHeading___Toc339612654)

[9.6 Safety Oversight (ISM plus SMC) 48](#__RefHeading___Toc339612655)

[10 Clinical Monitoring 50](#__RefHeading___Toc339612656)

[10.1 Site Monitoring Plan 50](#__RefHeading___Toc339612657)

[11 Statistical Considerations 51](#__RefHeading___Toc339612658)

[11.1 Introduction 51](#__RefHeading___Toc339612659)

[11.2 Overview and Study Objectives 51](#__RefHeading___Toc339612660)

[11.2.1 Primary Outcome Measures 51](#__RefHeading___Toc339612661)

[11.2.2 Secondary Outcome Measures 51](#__RefHeading___Toc339612662)

[11.3 Study Population 52](#__RefHeading___Toc339612663)

[11.4 Study Design 52](#__RefHeading___Toc339612664)

[11.5 Study Outcome Measures 53](#__RefHeading___Toc339612665)

[11.6 Study Hypotheses 54](#__RefHeading___Toc339612666)

[11.7 Sample Size Consideration 54](#__RefHeading___Toc339612667)

[11.8 Participant Enrollment and Follow-Up 54](#__RefHeading___Toc339612668)

[11.9 Planned Interim Analyses 55](#__RefHeading___Toc339612669)

[11.9.1 Safety Review 55](#__RefHeading___Toc339612670)

[11.9.2 Immunogenicity Analysis 56](#__RefHeading___Toc339612671)

[11.10 Final Analysis Plan 56](#__RefHeading___Toc339612672)

[11.10.1 Immunogenicity Analysis 56](#__RefHeading___Toc339612673)

[12 Source Documents and Access to Source Data/Documents 57](#__RefHeading___Toc339612674)

[13 Quality Control and Quality Assurance 59](#__RefHeading___Toc339612675)

[14 Ethics/Protection of Human Subjects 60](#__RefHeading___Toc339612677)

[14.1 Ethical Standard 60](#__RefHeading___Toc339612678)

[14.2 Institutional Review Board/Ethics Committee 60](#__RefHeading___Toc339612679)

[14.3 Informed Consent Process 61](#__RefHeading___Toc339612680)

[14.4 Exclusion of Women, Minorities, and Children (Special Populations) 61](#__RefHeading___Toc339612681)

[14.5 Participant Confidentiality 61](#__RefHeading___Toc339612682)

[14.6 Study Discontinuation 62](#__RefHeading___Toc339612683)

[14.7 Future Use of Stored Specimens 62](#__RefHeading___Toc339612684)

[15 Data Handling and Record Keeping 64](#__RefHeading___Toc339612685)

[Participant Confidentiality 64](#__RefHeading___Toc339612686)

[15.1 Data Management Responsibilities 65](#__RefHeading___Toc339612687)

[15.2 Data Capture Methods 65](#__RefHeading___Toc339612688)

[15.3 Types of Data 65](#__RefHeading___Toc339612689)

[15.4 Timing/Reports 65](#__RefHeading___Toc339612690)

[15.5 Study Records Retention 66](#__RefHeading___Toc339612691)

[15.6 Protocol Deviations 66](#__RefHeading___Toc339612692)

[16 Publication Policy 68](#__RefHeading___Toc339612693)

[17 Literature References 69](#__RefHeading___Toc339612694)

List of Abbreviations

| AE | Adverse Event/Adverse Experience |
| --- | --- |
| ALT | Alanine Aminotrasferase |
| ANA | Auto-Antibody screen |
| APSGN | Acute Post-Streptococcal Glomerulonephritis |
| ARF | Acute Rheumatic Fever |
| ASOT | Anti-streptolysin O titre |
| AST | Aspartate Aminotrasferase |
| CFU | Colony forming units |
| CI | Clinical Investigator |
| CIB | Clinical Investigator’s Brochure |
| CIOMS | Council for International Organizations of Medical Sciences |
| CPK | Creatine Phosphokinase |
| CRF | Case Report Form |
| CRP | C-Reactive Protein |
| CTMF | Clinical Trial Master File |
| CTC | Clinical Trial Coordinator |
| CTPC | Clinical Trial Protocol Committee |
| D | Diphtheria Toxoid |
| CRO | Contract Research Organization |
| DMID | Division of Microbiology and Infectious Diseases, NIAID |
| ECG | Electrocardiogram |
| ECHO | Transthoracic Echocardiogram |
| eCRF | Electronic Case Report Form |
| ELISA | Enzyme-Linked ImmunoSorbent Assay |
| ER | Emergency Room |
| EROA | Effective Regurgitant Orifice Area |
| FBC | Full Blood Count |
| FDA | Food and Drug Administration |
| GAS | Group A Streptococcus |
| GCP | Good Clinical Practice |
| GMT | Geometric Mean Titer |
| IATA | International Air Transport Association |
| ICF | Informed Consent Form |
| ICH | International Conference on Harmonisation |
| I.M. | Intramuscularly |
| IND | Investigational New Drug |
| IRB | Institutional Review Board |
| ISF | Investigator Site File |
| ISM | Independent Safety Monitor |
| IUC | Intra Uterine Contraceptive |
| J8 | Active peptide |
| Hb | Hemoglobin |
| HBsAg | Hepatitis B virus surface antigen |
| HCV | Hepatitis C virus |
| HREC | Human Research Ethics Committee |
| MCS | 6’-maleimido-caproyl n-hydroxy succinimide |
| MedDRA | Medical Dictionary for Regulatory Activities |
| MA | Memory Aid |
| N | Number (typically refers to subjects) |
| NATA | The National Association of Testing Authorities |
| NIAID | National Institute of Allergy and Infectious Diseases, NIH |
| NIH | National Institutes of Health |
| PBS | Phosphate Buffered Saline |
| PI | Program Investigator |
| PISA | Proximal Isovelocity Surface Area |
| PK | Pharmacokinetics |
| Plts | Platelet count |
| RAM | Regulatory Affairs Manager |
| RBWH | Royal Brisbane and Women Hospital |
| RF | Rheumatic Fever |
| RHD | Rheumatic Heart Disease |
| QA | Quality Assurance |
| QC | Quality Control |
| QIMR | Queensland Institute of Medical Research |
| SAE | Serious Adverse Event/Serious Adverse Experience |
| SDCC | Statistical and Data Coordinating Center |
| SMC | Safety Monitoring Committee |
| SOP | Standard Operating Procedure |
| TC | Telephone Call |
| TGA | Therapeutic Goods Administration |
| WBC | White Blood Cell |
| WHO | World Health Organization |

Protocol Summary

| **Title:** | A Randomized, Double Blinded Within Dose, Controlled, Safety and Immunogenicity of Group A Streptococcus Vaccine in Healthy Participants |
| --- | --- |
| **Phase:** | I |
| **Population:** | 20 healthy adults 18-45 years of age; Brisbane, Australia; The participants will be randomized to receive either investigational vaccine candidate or control saline at 0 and 8 weeks study product administration schedule. 15 participants will receive two injections containing 50µg of the vaccine candidate. Five participants will serve as controls and will receive two injections of saline at 8 weeks interval. |
| **Number of Sites:** | 1 |
| **Study Duration:** | 14 months |
| **Subject Participation Duration:** | 13 months |
| **Description of Agent or Intervention:** | The vaccine candidate is a synthesized peptide antigen from the conserved region of the M-protein (Peptide AcJ8) conjugated to Diphtheria Toxoid (D) as a carrier protein mixed with alum (Alhydrogel, 2% Aluminum hydroxide) adjuvant. The vaccine candidate will be formulated to contain 50µg of the peptide conjugate (15 µg of AcJ8 and 35 µg of D).It will be administrated intramuscularly (i.m.) in a total volume of 0.5mL. |
| **Objectives:** | Primary:   - To evaluate the safety of Group A Streptococcus vaccine candidate when delivered intramuscularly to healthy adults in two doses of 50μg AcJ8-D adsorbed onto alum.   Secondary:   - To assess the immunogenicity of a Group A streptococcus vaccine candidate delivered intramuscularly to healthy adults in two doses of 50 µg AcJ8-D adsorbed onto alum |
| **Description of Study Design:** | The purpose of this study is to evaluate the safety and immunogenicity of a Group A Streptococcus vaccine candidate. The participants (N=20) will be randomized 3:1 to receive the active intervention (N=15) or saline as placebo control (N=5). Participants randomized for intervention will receive either 50 µg of the vaccine candidate or saline. 2 participants will be dosed, 1 placebo and 1 investigational product 24 hours before the remaining 18 participants. They will be observed for 6 hours for any immediate adverse events. The ISM will review the data prior to the other 18 participants receiving study product. If no immediate serious adverse reactions have been observed the other 18 participants will receive an intramuscular injection of 0.5mL volume of the assigned study product. Safety of the first dose will be assessed by the Safety Monitoring Committee (SMC) before proceeding to administering the second dose to all 20 participants on Day 56. Clinical and adverse events/serious adverse event assessment will be conducted throughout the study at the scheduled clinic visits. Clinical specimens (urine and blood) will be collected from each participant to assess the safety and the immunogenicity of the investigational vaccine. Echocardiograms and electrocardiograms will be performed throughout the study to assess the safety of the vaccine formulation. |
| **Estimated Time to Complete Enrollment:** | The enrollment of all participants in this study will take approximately 4 weeks. |

**Schematic of Study Design**: Screening, Study Product Administration, Safety Testing, Safety Monitoring Committee Review for the cohort of 20 subjects

| **Vaccine Trial Schedule** | |
| --- | --- |
| **Time** (day) |  |
| Day -28 to Day -7 | Screening  ECG/ECHO/hematology/chemistry/urinalysis/ASOT/ α-DNAse B |
| Day 0^ | Dose 1 (n = 15A*, 5C*) |
| Day 8±2 | Memory aid completion |
|  |  |
| Day 28±7 | ECG/ECHO/hematology/chemistry/urinalysis/ASOT/α-DNAse B |
| Day 35±5 | Database Collection of Data (up to Day 28 data) |
|  |  |
| Day 49±5 | SMC Dose 1 review |
| Day 56±5 | Dose 2 (n = 15A*, 5C*) |
| Day 64±2 | Memory aid completion |
|  |  |
| Day 84±7 | ECG/ECHO/hematology/chemistry/urinalysis/ASOT/α-DNaseB |
| Day 98±5 | Database Collection of Data (up to Day 84 data) |
|  |  |
| Day 126±7 | SMC Dose 2 review |
|  |  |
| Day 180±14 | Follow up visit  Hematology/chemistry/urinalysis/ASOT/ α-DNAse B |
|  |  |
| Day 266±14 | Follow up visit  Hematology/chemistry/urinalysis/ASOT/ α-DNAse B |
|  |  |
| Day 350±14 | Final follow up visit  ECG/ECHO/hematology/chemistry/urinalysis/ASOT/ α-DNaseB |

A* subjects receiving the interventional vaccine candidate

C* subjects receiving saline,

^ Two participants randomized to receive 1 active vaccine (A) and 1control saline (C) will be dosed 24 hours prior to remaining 18 participants.

A detailed schematic description of all visits and assessments is included in Appendix A: Schedule of Procedures/Evaluations.

# Key Roles

| **Individuals:**  **Sponsor** | **Clinical Investigator:**  Dr James S. McCarthy, M.D. FRACP  (Authority signatory on Protocol)  Queensland Institute of Medical Research Infectious Diseases and Immunology 300 Herston Road, Herston 4006 Brisbane, Queensland, Australia Phone: 61-7- 3845 3796 Fax: 61-7-3362 0104 E-mail: James.McCarthy@qimr.edu.au  **Program Investigator:**  Michael F. Good, MD PhD FRACP (Hon) FQA FTSE  Institute for Glycomics G26/4.18 Gold Coast Campus Griffith University  QLD  4222 Phone: +61 7 5552 9435 Fax: +61 7 5552 8098 E-mail: michael.good@griffith.edu.au  **Co-Investigators:**  Dr. Paul Griffin MBBS FRACP FRCPA.  Q-Pharm Pty. Ltd.  Clive Berghofer Cancer Research Centre (CBCRC)  300 Herston Road, Herston 4006 Brisbane, Queensland, Australia Phone: 61-7- 3845 3647 Fax: 61-7- 3845 3637  Dr. William Parsonage,  Cardiologist  Department of Cardiology,  Level 3, James Mayne Building,  Royal Brisbane and Women’s Hospital  Herston 4029, Brisbane, Queensland, Australia Phone: 61-0422234238 Fax: 61-7- E-mail: william_parsonage@health.qld.gov.au  **Statistician:**  Dr Peter O’Rourke  Queensland Institute of Medical Research 300 Herston Road, Herston 4006 Brisbane, Queensland, Australia Phone: 61-7-3845 3579 Fax: 61-7-3362 0110 E-mail: [Peter.ORourke@qimr.edu.au](mailto:Peter.ORourke@qimr.edu.au)  **Independent Safety Monitor**  Anthony Allworth MBBS FRACP  Infectious Diseases Unit, Royal Brisbane & Women's Hospital, Bowen Bridge Road Herston, Queensland Australia 4029, Australia Phone: 61-7- , 61-7- 3636 8761 Fax:61-7-,3636 1388 E-mail: Tony_Allworth@health.qld.gov.au  **Queensland Institute of Medical Research**  300 Herston Road, Herston 4006 Brisbane, Queensland, Australia Contact Person: Joy Black Phone Number: 61-7-3362 0203 Fax Number: 61-7-3362 0110 E-mail: [Joy.Black@qimr.edu.au](mailto:Joy.Black@qimr.edu.au) |
| --- | --- |
| Authorized Sponsor Signatory  Monitor’s Sponsors | Professor Frank Gannon, Director QIMR  Clinical Network Services |
| **Institutional Ethic Committee to which Q-Pharm is responsible:**  **Contract Research Organizations:** | **Queensland Institute of Medical Research Human Research Ethics Committee (QIMR-HREC):**  PO Box Royal Brisbane and Women’s Hospital  Herston 4029  Brisbane, Queensland, Australia HREC Admin Officer  Phone Number: 61-7-3362 0117 Fax Number: 61-7-3362 0109 E-mail: [HREC.Secretariat@qimr.edu.au](mailto:HREC.Secretariat@qimr.edu.au)  Q-Pharm Pty. Ltd  Terry Hurst,  Chief Executive Officer  Q-Pharm Pty. Ltd. Clive Berghofer Cancer Research Centre (CBCRC) Herston Road, Herston 4006 Brisbane, Queensland, Australia Phone: 61-7- 3845 3704 Fax: 61-7- 3845 3637 E-mail: t.hurst@qpharm.com.au |
| **Clinical Laboratory** | Sullivan Nicolaides Pathology   | PO Box 344 Indooroopilly, QLD 4068 Australia |  | | | --- | --- | --- | | Phone: 07 3377 8666 |  |  | | Fax: 07 3377 8722 |  |  | |  | | | |  | | | |
|  | The Childrens Hospital at Westmead  Hawkesbury Road  Westmead NSW 2145 Phone: (02) 9845 0000 |
|  | Institute for Glycomics (G26)  Gold Coast Campus  Griffith University  Queensland 4222  Australia  Phone: 07 5552 9434 |

# Background Information and Scientific Rationale

## Background Information

Group A streptococcus (GAS) is a serious human pathogen affecting people of different ages and all socio-economic levels. Pathology due to GAS can be divided into acute suppuration and post-streptococcal sequelae. The former include the common streptococcal pharyngitis and pyoderma, particularly common in children, and the far more serious necrotizing fasciitis, pneumonia, and toxic shock syndrome, primarily affecting older individuals. The post-streptococcal sequelae include rheumatic fever (RF), rheumatic heart disease (RHD) and acute-glomerulonephritis. RHD is of the most concern as it can lead to heart failure and a significantly shortened lifespan. The disease is confined largely to Indigenous populations of developed countries and developing countries where over-crowding and poor access to health care are contributing factors.

In the World Health Organization (WHO) World Health Report 2000, it was estimated that global RHD mortality in 1999 was approximately 376,000 deaths with the majority of these in the WHO regions of South-East Asia and the Western Pacific [5]. A recent review of the global burden of streptococcal diseases [6, 7] estimated that there are approximately 15.6 million existing cases of RHD plus 460,000 new RHD cases and 349,000 RHD-related deaths each year. Moreover, it was also estimated that there are approximately 663,000 new cases of invasive GAS disease each year resulting in 163,000 deaths. The majority of these RHD and invasive GAS disease cases occur in less developed countries. The global burden of non-invasive GAS associated diseases, such as pyoderma and pharyngitis, was also estimated at 111 million current cases of pyoderma and 616 million new cases of pharyngitis each year [6, 7].

For example, Australia's Aboriginal population experiences the highest documented rate of the disease in the world, with the incidence of RF being as high as 651 per 100,000 per year, and the prevalence of RHD being as high as 30 per 1000 [8]. The average age of onset of RF in Aboriginal children is 11 years and the mean life expectancy of Aborigines with RHD is 33 years, reviewed in [9]. Since RF follows infection with group A streptococci, a practical strategy to prevent the disease is to prevent streptococcal infection. These data, taken together with the dynamic epidemiology of GAS in combination with episodic resurgence of GAS virulence, highlight the need for a prophylactic vaccine.

Our research has primarily focused on the M-protein, which has a helical structure and exists as a ‘coiled-coil’ protein. The sequences of different M-proteins are highly variable at the amino terminal end of the molecule, but become highly conserved (>98% sequence identity) towards the carboxyl terminus. The M-protein determines the serotype of the organism, and the serotypic determinants are located at the amino terminus of the M protein. Antibodies directed to the serotypic determinants are able to opsonise the specific organism in the presence of neutrophils.

The hyper-variable N-terminal region of the M-protein has been shown to be highly immunogenic and induces a strain-specific immunity. Therefore, several different approaches have been used to combine different N-terminal epitopes together as one vaccine construct. Substantial progress has been made with a recombinant multi-valent protein vaccine that consists of multiple N-terminal epitopes of the M-protein from different GAS strains (emm types). Initial studies utilized a hexavalent construct [10]; however, more recently the number of epitopes has been expanded to increase the vaccine coverage for predominant GAS strains found in the USA [11]. In a recent study, this 26-valent vaccine candidate was found to induce antibodies that could opsonise not only the specific M-type represented in the vaccine but also sub-type variants that can have small differences in amino acid identity, indicating that the 26-valent vaccine could opsonise sub-type variants that may arise in a highly immunised population [12]. These studies also demonstrated that the 26-valent vaccine candidate was well-tolerated when administered in Phase I and Phase II human clinical trials [13].

**Previous GAS Vaccine Development**

Vaccine clinical trials in humans to prevent RF were conducted even before its etiology as a post-streptococcal infection was clearly defined in 1931. For example, in 1906, a vaccine was produced from streptococcal bacteria isolated from scarlet fever participants. This vaccine was subsequently administered to children in Europe [14, 15]. However, once Streptococcus pyogenes was identified as the organism responsible for RF and RHD, vaccine research focused on the M-protein. During the 1960s and 1970s multiple studies were conducted using purified M-protein or M-protein derivates in adults [16, 17] and children [18, 19]. These studies demonstrated that the vaccines based on purified M protein as either whole molecule or partially digested were able to induce production of type M protein specific antibodies. In a study in which 21 healthy siblings of patients with rheumatic fever where administered a partially purified M protein vaccine in doses that ranged from 0.35mg to 6mg in a series of 18 to 33 weekly subcutaneous injections [18, 20], significant local and systemic reactions were observed [reviewed in [21]. Further, the frequency of GAS infection appeared to be higher among those children given the vaccine, compared to their unvaccinated siblings, and definite RF occurred in 2 out of 21 subjects during the study, albeit likely due to intercurrent GAS infection with a different serotype rather than as a direct consequence of the immune response to the M protein [20]. Although the association between the induction of a antibodies cross reacting with heart tissue by streptococcal infection and the development of ARF and RHD have been described [22] this remains controversial as a cause and effect relationship remains to be established and human cross-reactive antibodies have not been reported in participants of any previous vaccine clinical trial (see below).

Of note, RF has not been observed in association with most M protein-based vaccines. For example, studies involving partially digested M protein administrated at dosages ranging from 800 µg to 1100 µg in a series of 4 to 7 injections, no serious local or systemic responses were observed. Moreover, none of the participants developed antibodies that cross reacted with heart tissue [16]. In a further series of studies using a highly purified M protein-based vaccine administrated in both adults and infants [17, 20], no cross-reactive antibodies and no cases of RF were reported [10, 13]. These findings triggered research aimed at identification of an M protein epitope capable of eliciting specific immunogenicity without the risk of development of cross reactive antibodies that potentially could contribute towards the pathology of the RHD.

**The Conserved Region Approach to GAS Vaccine Development**

The highly conserved C-repeat region has come to prominence as a possible area to be targeted for vaccine design largely because of the difficulties in defining common N-terminal epitopes that could lead to a vaccine covering all regions of the world. In 1992 and 1994, we published articles [23, 24] in The Journal of Immunology and in The Lancet describing a 20-mer peptide (p145) that induced antibodies in vaccinated mice that opsonised multiple serotypes of GAS. We also showed that the prevalence of p145-specific antibodies amongst humans living in streptococcal-endemic areas increased with age, being found in approximately 40% of children and over 90% of adults [25] - increasing in parallel with the acquisition of immunity to group A streptococci.

We also demonstrated that affinity purified human antibodies to p145 could directly opsonise multiple strains of GAS in vitro [25, 26]. We have sequenced the conserved region of multiple GAS strains from Australia, Thailand, USA and India and observed only two variants in the p145 region of the M protein. These two variant sequences are immunologically cross-reactive and peptide-specific antibodies from B10.BR mice can opsonise and kill in vitro GAS bearing either p145 sequence. Peptide 145 would be a vaccine candidate except for the fact that human T cells specific for p145 can react with human heart tissue in vitro [27]. Even though these T-cells were derived from an individual without any evidence of heart disease, there was a possibility that an immune response induced by p145 could induce disease. We therefore determined the minimal antibody epitope on the peptide and separated this epitope from the detrimental T-cell epitope. Since we previously determined that p145 was a conformational epitope with alpha helical propensity [28], a technique was developed to enable us to map the epitope by embedding small peptides from p145 within other larger and unrelated peptides derived from GCN4 (a DNA-binding protein of yeast), which were known to also form a helical coil.

These peptides (Figure 1), referred to as chimeric peptides (J1 to J9), maintained the appropriate tertiary structure, and enabled us to map the minimal antibody epitope and separate it from a T cell epitope. The antibody epitopes recognised by endemic human sera were chimeric peptides J2, J7 and J8 [25], whereas the chimeric peptides recognized by murine antisera were J7 and J8 [28, 29]. Human T-cells respond to J2 and murine T-cells from B10.BR mice immunised with p145 respond vigorously to J3 [29]. B10.BR (H-2k) mice have been immunized with J8 in Complete Freund’s adjuvant and have produced antibodies capable of opsonising different strains of GAS. These vaccinated mice were also protected following GAS challenge with virulent organisms.

p145 LRRDLDASREAKKQVEKALE

J1 QLEDKVKQLRRDLDASREAKEELQDKVK

J2 LEDKVKQARRDLDASREAKKELQDKVKQ

J3 EDKVKQAERDLDASREAKKQLQDKVKQL

J4 DKVKQAEDDLDASREAKKQVQDKVKQLE

J5 KVKQAEDKLDASREAKKQVEDKVKQLED

J6 VKQAEDKVDASREAKKQVEKKVKQLEDK

J7 KQAEDKVKASREAKKQVEKAVKQLEDKV

J8 QAEDKVKQSREAKKQVEKALKQLEDKVQ

J9 AEDKVKQLREAKKQVEKALEQLEDKVQL

J14 KQAEDKVKASREAKKQVEKALEQLEDKVK

**Figure 1:** Chimeric peptides with M protein derived sequence in bold text and the flanking regions derived from the yeast protein GCN4 in normal text.

**The Vaccine Candidate**

Preclinical immunization and challenge studies [30] were used to investigate the immunogenicity and protective potential of a novel M protein epitope i.e. J8 peptide based vaccine formulation. J8 peptide has been conjugated to the classic vaccine molecule and carrier protein, diphtheria toxoid (D), to create an immunogen that can induce antibodies in an outbred population in mice. D has been used as a carrier protein in many systems, and preliminary data from our own laboratory suggested that anti-D antibodies may in fact be capable of partially opsonising GAS, making D an obvious choice for a carrier protein for J8. The peptide was synthesized with an additional Cys residue on the carboxyl terminus, which enabled the conjugation to the D using 6’-maleimido-caproyl n-hydroxy succinimide (MCS) chemistry. The vaccine was administered with the human compatible adjuvant, alum.

It was found that the J8-D/alum formulations induced J8-specific serum IgG antibodies in both the inbred B10.BR (H-2k) and outbred Quackenbush mouse strains [30]. Namely, the inbred and outbred mice immunized subcutaneously with J8-D/alum developed significantly (p<0.05) higher J8-specific serum IgG titers compared to cohorts of mice immunized with either D/alum or PBS/alum. The average opsonic activity of sera collected from the J8-D immunized groups ranged from 49.2% to 87.3% and were significantly greater than sera from the D and PBS immunized groups (p<0.05).

In addition, groups of mice immunized with J8-D/alum that developed J8-specific serum IgG also had significantly (p<0.05) increased numbers of mice surviving following an intraperitoneal challenge with GAS strains (including M1, M6 and the clinical isolate 88/30), compared to control groups immunized with D/alum or PBS/alum (<0.05) for each of the GAS strains tested. These results indicated a positive correlation between J8-specific serum IgG and survival, indicating the importance of IgG in survival.

The antigen in the proposed vaccine candidate is the J8 peptide, 29 amino acid (aa) long peptide from the conserved region of the M-protein found on the surface of group A streptococci. This peptide, is further embedded in a flanking region derived from GCN4 (a DNA-binding protein of yeast) to retain the helical coil structure of the antigen, resulting in the J8 chimeric vaccine construct.

However during development, prior to the manufacture for the toxicology study and clinical trial, it was discovered that N terminal glutamine (Gln) residue spontaneously cyclizes and converts into pyroglutamic acid (pGln). The process of deamidation of the Gln to pGln was suspected to continue even when the peptide was conjugated to the diphtheria toxoid carrier molecule. Animal studies were conducted and confirmed that the transformation of Gln to pGlu did not affect the immunogenicity of the vaccine formulation, which confirmed that Gln and pGln do not differ biologically. Animal data supporting this finding will be provided in the IND submission. For the purpose of having a uniform species of the peptide for the GMP product, the peptide was deamidated, a process in which all N-terminal Gln residues were converted into pGln and then conjugated to the carrier protein. The deaminated J8 peptide is referred as acetylated J8 (AcJ8) peptide and the final vaccine formulation consists of acetylated J8 peptide conjugated to diphtheria toxoid (AcJ8-D).

The proposed vaccine formulation contains 50μg of AcJ8-D conjugate (approximately 15 µg of J8 and 35 µg of diphtheria toxoid) adsorbed to Alhydrogel 2% (2% Aluminum hydroxide, alum).

The present study is being performed to investigate the safety of this novel vaccine formulation and the ability of this vaccine to induce an immune response in humans. The present study will be conducted in healthy adult participants from Brisbane, Australia. This study will be conducted in compliance with the protocol, ICH GCP and TGA regulatory requirements.

## Rationale

Preclinical studies have indicated that induction of antigen-specific serum IgG is important in protecting against lethal GAS challenge. We have previously demonstrated a significant positive correlation between J8-specific serum IgG titers in both inbred and outbred mice and survival following GAS challenge [30]. To expand on this work we conducted passive transfer experiments that demonstrated that hyperimmune sera from donor mice immunized with J8-D/alum could protect naive recipient mice from lethal GAS challenge. In addition, purified IgG from rabbits immunized with J8-D/alum was also capable of protecting mice from GAS challenge (unpublished).

With this in mind, we then investigated the serum IgG responses in mice administered increasing doses of vaccine formulation. There was a significant increase in the level of antigen-specific serum IgG induced by 30 μg of J8-D compared to only 3 μg [30]. This increase in antibody titer corresponded with an increase in the percentage of mice surviving post-challenge. In general, this trend of increasing IgG titers was consistent when higher doses of J8-D were administered; however, the increase in average antigen-specific serum IgG titer was not significant, possibly due to the non-linear response to the antigen at high doses such as 500 µg (unpublished). Additional immunogenicity studies in mice have however indicated that a vaccine regimen that consisted of two doses of 50 µg of peptide-conjugate antigen formulated with adjuvant is sufficient to trigger immune response. The observed geometric mean titer (GMT) of peptide specific antibodies in mice was high and remained high 10 weeks after the last immunization.

The J8-D vaccine formulations were originally administered subcutaneously in mice. However, recent studies in mice have indicated that there is no significant difference in average antigen-specific serum IgG titers when the formulation is administered subcutaneously or intramuscularly (unpublished). However, preliminary data have indicated that HepB absorbed with alum induces a higher IgG titer when administered intramuscularly compared to subcutaneously (personal communication).

Pittman and colleagues demonstrated that reaction rates to an alum absorbed Anthrax vaccine decreased when the time interval between the first and second injection was increased from 2 to 4 weeks [31]. Moreover, erythema and induration were significantly more common in subjects who were administered the vaccine subcutaneously compared to intramuscularly. Similarly, the duration and intensity of redness, swelling, itching and pain were significantly reduced in adolescents administered D intramuscularly compared to subcutaneous administration [32].

Vaccines that contain alum adjuvant such as DTP, Hepatitis A, Boostrix, and Adacel are administrated intramuscularly. Moreover, Menactra, a vaccine licensed in the U.S. that is formulated to contain diphtheria toxoid as a carrier molecule at approximately the same concentration as that in our vaccine candidate is also administrated intramuscularly. These findings justify that intramuscular administration is the preferable delivery option for the formulation proposed for our investigational GAS vaccine. The current proposed clinical trial protocol has been developed in consideration of these data.

Based on review of all the findings it was concluded that, to achieve an optimal immunogenic response in human, the vaccine formulation should contain 1x peptide-carrier ratio which will be formulated by adsorption to alum and it will be administrated in total of two doses.

We hypothesize that the vaccine formulation will be safe, immunogenic in humans and induce an antigen-specific systemic serum IgG response.

## Potential Risks and Benefits

### Potential Risks

No potential risks have been identified in preclinical studies of the investigational vaccine candidate in the course of the animal work performed using different vaccine formulations. However, due to the fact that the J8 peptide is part of the M-protein, there exists the potential of inducing local and systemic reactions as reported in other studies that involved investigational group A streptococcus vaccines based on the M protein. These include local reactions such as mild to moderate erythema accompanied by swelling and tenderness at the injection site. Another possible risk is the induction of a human cross reactive (autoimmune) antibody response of a similar quality that may lead to the pathology observed in RHD and RF in the study participants. However, safety monitoring will be included throughout the study. Serum screening using rabbits for cross reactive antibodies in the vaccinated participants in GAS vaccine studies has been reported previously [13]. However it has been shown that this assay is limited in demonstrating the safety of the vaccine in respect to induction of cross reactive antibodies. At present, there is no sufficiently well developed and reliable assay that could be found in non-human primates [37-43], thus serum samples will be kept for each participant at time points indicated in this protocol and tested should better assay become available.

Possible risks could also include allergic reaction to the vaccine components or reactions reported with the administration of vaccines containing diphtheria toxoid conjugate in their composition.

These reactions include headache, localized pain, redness and swelling at injection site, fever, fatigue, anorexia, diarrhea and less commonly reported reactions such as vomiting and rash.

Other possible risks would also include the risks associated with the process of obtaining clinical samples e.g. (blood) i.e. venipuncture. These risks would include mild discomfort due to venipuncture, swelling, bleeding at the site of venipuncture, and bruising at the puncture site. Light headedness/fainting could also occur at the time of the blood collection, but this is uncommon and on rare occasions, infections due to the venipuncture. In accordance to the clinical site procedures, standard precautions will be taken to reduce the likelihood of these difficulties.

### Known Potential Benefits

There are no known direct benefits to the participants receiving the Group A streptococcus vaccine. There will be benefit to the community by contributing to research for the development a group A streptococcus vaccine. There may be a benefit to the participant from the results of the screening tests and procedures (blood tests, electrocardiogram (ECG) and transthoracic echocardiograms (ECHO)).

# Objectives

## Study Objectives

**Primary objectives:**

To evaluate the safety of a Group A streptococcus vaccine candidate delivered intramuscularly to healthy adults in two doses of 50 µg of AcJ8-D adsorbed onto alum.

**Secondary objective:**

To assess the immunogenicity of a Group A streptococcus vaccine candidate delivered intramuscularly to healthy adults in two doses of 50 µg AcJ8-D adsorbed onto alum.

## Study Outcome Measures

### Primary Outcome Measures

The safety of the vaccine candidate will be assessed on the basis of the frequency and occurrence of injection site and systemic reactions following administration of the investigational vaccine. Adverse events (AE) and serious adverse events (SAE) will be reported throughout the duration of the study. Safety parameters that will be monitored include changes in physical examination including cardiac auscultation, clinical biochemistry and hematology testing on blood and urine, and changes in electrocardiogram or transthoracic echocardiograms.

### Secondary Outcome Measures

• The production and the titer of the vaccine specific antibodies of the baseline titer after study product administration will be determined using ELISA. The presence of vaccine-specific antibodies in all participants in the study will be monitored and the titer of the vaccine specific antibodies after each dose of the vaccine candidate will be measured to investigate the relationship between the doses administered and the titer of vaccine-specific antibodies.

• The bactericidal activity of the induced antibodies against different standard reference strains of group A streptococcus such as M1, M6 and 88/30 (Australian clinical isolate) will also be assessed.

# Study Design

This study is a double blinded, randomized Phase I single cohort clinical study (N = 20 participants). The participants will be randomized 3:1 to receive the intervention (N=15) or control (saline), (N=5). Each participant will receive a total of two injections with an 8-week interval between injections.

To investigate for the occurrence of any immediate serious adverse reactions to the injected vaccine candidate, two (2) participants (one randomized to receive the study vaccine and one to receive the control saline) will have study product administered 24 hours before the remaining eighteen (18) participants. These two participants will be observed for at least 6 hours after study product administration to evaluate that no immediate serious adverse reactions have occurred, by measuring vital signs, change in the baseline symptoms and monitoring for occurrence of clinically significant symptoms and adverse events, The clinical investigator (CI) and the Independent Safety Monitor (ISM) will review all reactions including the solicited events that have occurred within the observation window for these two participants following study product administration and decide on whether the remaining participants will receive the first dose. If no immediate serious adverse reactions have been observed, the remaining participants will receive the first dose. The clinical and the adverse events recorded at the scheduled interim visits will be assessed by the SMC to assess the safety of the vaccine candidate prior to proceeding to the second dose.

Throat swabs will be taken from each participant at screening and on each day of study product administration. Rapid antigen test (QuickVue In-Line Strep A test) for group A streptococci will also be performed for each participant prior to each study product administration. Targeted physical examinations including cardiac auscultation by a physician will be performed on the day of each study product administration. Each participant will be observed for 30 minutes following administration of study product for evidence of immediate reactions.

A Memory Aid (MA) will be provided to participants to record their oral temperature, solicited and unsolicited symptoms, adverse events and concomitant medications. Each participant will be asked to record in memory aids the occurrence of any symptoms at the injection site (such as pain, tenderness, pruritis, erythema and induration) and systemic reactions such as fever, anorexia, nausea, malaise, myalgia, arthralgia and headache. Participants will also be asked to record any changes in urine that might suggest haematuria (blood-tinged or cola-coloured urine) and cutaneous abnormalities (rash or subcutaneous nodules). Information will be recorded daily for 7 days after each injection. Study personnel will review the information on the Memory Aid with the participant via phone calls (day 2 and 8 days after injection) to elicit as much information as possible about any reported symptoms. Based on this information, study staff will use his/her clinical judgment to assess the event and its severity and record the data on the Reactogenicity Record. Study staff will use the Reactogenicity Record to enter information into the database. The Memory Aid will not be collected from the participants.

All participants will be encouraged to contact clinical investigators or to visit the clinical site if they observe any grade 3 AE of the above mentioned reactions following each immunization.(see Appendix B), or if they have any concern. Adverse events and serious adverse events will also be reported at clinic visits throughout the study period.

Each participant will be required to visit the investigation site at the allocated days. Targeted physical examinations including cardiac auscultation by physician will be performed on day 28 after each vaccination and at follow-up visits on days 180, 266 and 350. Additional cardiac evaluation will include an electrocardiogram and transthoracic echocardiogram at day 28 following each injection and at the final follow-up visit on day 350. All study visits will be within the acceptable windows shown on Appendix A.

Biochemical and hematologic analyses for safety parameters including urinalysis will be performed for all participants at screening, prior to each injection and then at day 28 after each administered dose and at follow-up visits on days 180, 266 and 350. A urine pregnancy test will also be performed for all female participants on the day of each vaccination. Immunogenicity of the vaccine components will be determined by measuring the serum level of GAS peptide specific antibodies and cellular responses on each day of vaccination 28 days after each vaccination and at follow-up visits on days 180, 266 and 350. The induced level of GAS peptide antibodies will be used to monitor the kinetics of the antibody response after two vaccinations and to evaluate the response of subjects receiving the interventional vaccine compared to the placebo saline control. Moreover, serum sample of each participant at time points prior to each injection and at the final study visit on day 350 will be kept for use in testing for presence of human tissue cross-reactive antibodies should suitable assays for detection of cross reactive become available. The level of the anti-diphtheria toxoid antibodies in each participant will be measured prior to the each injection and at the end of the study. The expected duration of participant’s participation from screening to the final follow up visit will be approximately 13 months.

# Study Enrollment and Withdrawal

This study will enroll 20 healthy adults between 18-45 years of age. Both females and males will be eligible to enroll in the study. No restrictions will apply for ethnic or racial categories. The expected population to be enrolled may include all Australian racial categories, such as Australian White, Australian Indian, Australian Asian, Australian Aborigines or Torres Strait Islanders. This study will be conducted at Q-Pharm Pty. Ltd, Brisbane Australia. Q-Pharm is an independently registered Company that conducts Phase I clinical trials. It is co-located on the campus of the Royal Brisbane and Women’s Hospital (RBWH) and QIMR. Q-Pharm also offers services for participant recruitment for clinical studies from a variety of sources. Prior to their use, all advertisements used for recruitment of the participants in the study will be approved by the ethics committees of all institutions concerned. The enrollment of participants in the study will be performed in accordance with the principles of the ICH E6 Good Clinical Practice [3]. Screening consent will be obtained from each participant to collect the baseline specifications that will indicate the suitability of the participants for inclusion in this study. Once the suitability of the participants is confirmed at the screening visit, consent will be obtained from each participant to enroll into the interventional part of this study. Alternates may also be identified and held in reserve. The reserve participants will be asked to attend the clinic on Day 0, but they will take a part in the study if one or more participants withdraw before the first injection of the study vaccine.

## Participant Inclusion Criteria

All participants in the clinical study must meet all of the following inclusion criteria to participate in this study:

• Able to understand the purpose and the procedures involved in this study and sign the informed consent form;

• Male or non-pregnant female adults, 18-45 years of age inclusive;

• Non smokers and in good general health, as determined by screening evaluation, no greater than 28 days before the first dose in the form of medical history, clinical laboratory tests and physical examination;

• Normal Electrocardiogram (ECG);

• Echocardiogram (ECHO) that is normal or with findings that are considered trivial and clinically insignificant such as:

- 'Clinically insignificant/trivial mitral regurgitation (EROA of <10mm2, quantified as described in Appendix H) ;

• Women must agree not to become pregnant for the first 180 days of the trial. If they are sexually active, they must use an effective method of birth control, e.g. insertable, injectable, transdermal, or combination oral contraceptive approved by the US FDA or TGA combined with a barrier contraceptive and have negative results on a serum or urine pregnancy test done before administration of study medication;

• Intention to reside in the geographical area for next 12 months and not intending to travel overseas for at least 30 days following the last study vaccine administration;

• Agree not to participate in any other clinical trial during the trial;

• Agree not to donate blood for the first 180 days of the trial;

• Agree to restrain from intensive physical exercise i.e. exercise that varies significantly from an every day exercise routine, 3 days before and after (± 3 days) administration of each dose, including each interim visit for blood sample collection.

## Participant Exclusion Criteria

Any potential participants meeting any of the following criteria will be excluded from the study:

• Personal or family history of post-streptococcal disease (rheumatic fever or glomerulonephritis), or collagen-vascular disease;

• Has evidence of increased cardiovascular disease risk (defined as >10%, 5 year risk) as determined by the method of Gaziano et al., (36). Risk factors include sex, age, systolic blood pressure (mm Hg), smoking status, body mass index (BMI, kg/m^2^), reported diabetes status and blood pressure;

• Clinical diagnosis or evidence of recent group A streptococcal infection as measured by anti-streptolysin O or anti-DNase B levels exceeding 200 units;

• Positive group A streptococcus throat culture at screening visit or positive rapid antigen test result on day of study product administration;

• Evidence of multiple past group A streptococcus infections as measured by anti-J8 antibodies levels > 3 Standard Deviations above background Optical Density;

• Presence of significant acute infections requiring systemic antibiotic treatment within the 14 days prior to each product administration;

• Pregnant or breast feeding (all women will have a negative pregnancy test result prior to each study product administered);

• Immunized or intent to immunize with any vaccine or investigational agents within 30 days prior to enrollment through to 30 days following the last study vaccine administration, with the exception of licensed inactivated influenza vaccines;

• Receipt of diphtheria toxoid containing vaccine within the previous 5 years (such as DTP, Hepatitis A, Boostrix, Adacel or Menactra);

• Past significant reaction following any previous vaccination;

• History of hypersensitivity to any diphtheria toxoid containing vaccine;

• Presence of acute infectious disease or fever (e.g., sub-lingual temperature ≥ 38.5°C) within the five days prior to study product administration;

• Presence of current or suspected serious chronic diseases such as cardiac or autoimmune disease (HIV or other immunodeficiencies), insulin dependent diabetes, progressive neurological disease, severe malnutrition, acute or progressive hepatic disease, acute or progressive renal disease, psoriasis, rheumatoid arthritis, asthma, epilepsy or obsessive compulsive disorder, skin carcinoma excluding non spreadable skin cancers such as basal cell and squamous cell carcinoma;

• Evidence and any history of leukaemia, lymphoma or neoplasm;

• Presence or suspicion of impaired immune system function. Currently receiving or having within the past three years received immunosuppressive therapy, including systemic steroids, ACTH or inhaled steroids in dosages that are associated with hypothalamic-pituitary-adrenal axis suppression, such as 1mg/kg/day of prednisone or its equivalent or chronic use of inhaled high potency corticosteroids [budesonide 800 µg per day or fluticasone 750 µg];

• Received blood, blood products or a parenteral immunoglobulin preparation in the past 12 weeks;

• Evidence of bleeding diathesis or any condition that may be associated with a prolonged bleeding time;

• Known inherited genetic anomaly (known as cytogenic disorders) e.g., Down’s syndrome;

• Evidence of any condition that, in the opinion of the clinical investigator, might interfere with the evaluation of the study objectives or pose excessive risks to participants;

• Findings of definite, probable or possible rheumatic heart disease (RHD), definite or probable acute rheumatic fever (ARF) as defined in appendix F [35];

• Echocardiographic findings such as:

- - Cardiac Chambers: left ventricular dilatation (based on LV diameter > reference ranges for LV end diastolic diameter (LVEDd) indexed for body surface area (Females 24-32 mm/sqm; Males 22-31mm/sqm);
  - left ventricular dysfunction (Ejection Fraction < 50%; left ventricular hypertrophy (LV wall thickness > 11mm); Right ventricular dysfunction or dilatation (Subjective assessment);
  - Cardiac Valves/Haemodynamic Findings: Clinically significant mitral regurgitation defined: at the discretion of the cardiologist and/or effective regurgitant orifice area of ≥10mm^2^; Any degree of valvular stenosis or left ventricular outflow tract obstruction; Pulmonary hypertension (defined as an estimated right ventricular systolic pressure of >40 mmHg, calculated using the peak tricuspid regurgitant jet velocity method);
  - Any aortic regurgitation;
- Pericardium: greater than trivial pericardial fluid (trivial defined as < 5mm and not circumferential);
- Pre-existing significant structural valve disease (for example, but not limited to bicuspid aortic valve regardless of haemodynamic effect, mitral valve prolapse regardless of severity of regurgitation, pulmonary stenosis);
- Other significant congenital lesions (for example, but not limited to aortic coarctation, septal defect, excluding patent foramen ovale (NOTE: findings considered normal developmental variation, specifically including patient foramen ovale and prominent Eustachian valve will not be considered exclusion criteria;

• Clinical or sub-clinical acute post-streptococcal glomerulonephritis (APSGN), as defined in appendix F [35];

• Clinical significant abnormal laboratory results e.g., CBC with differential and platelets, AST, ALT, total bilirubin, urea, creatinine, electrolytes (including sodium, potassium, chloride and bicarbonate), troponin T, C-reactive protein, C3 complement, Antinuclear antibodies (>320 titer or >160 with homogeneous pattern) and anti-cyclic citrullinated peptide antibodies (Anti-CCP antibody);

- The participant has a diagnosis of schizophrenia, bi-polar disease, or other severe (disabling) chronic psychiatric diagnosis;
- The participant has been hospitalized within the past 5 years prior to enrollment for psychiatric illness, history of suicide attempt or confinement for danger to self or others;
- The participant is receiving psychiatric drugs^1^. Participants who are receiving a single antidepressant drug and are stable for at least 3 months prior to enrollment without decompensating are allowed enrollment into the study;

^2 1^aripiprazole, clozapine, ziprasidone, haloperidol, molindone, loxapine, thioridazine, thiothixene, pimozide, fluphenazine, risperidone, mesoridazine, quetiapine, trifluoperazine, trifluopromazine, chlorprothixene, chlorpromazine, perphenazine, olanzapine, carbamazepine, divalproex sodium, lithium carbonate or lithium citrate.

- The participant has a history of alcohol or drug abuse in the 5 years prior to enrollment.

## Treatment Assignment Procedures

### Randomization Procedures

Enrollment will be done by Q-Pharm. The randomization code will be included in the enrollment module for the trial. The randomization code will link the vial allocation number to the treatment assignment. Each subject enrolled in the trial is assigned to a vial allocation number after demographic and eligibility data have been entered into the system. The code list for emergency unblinding purposes will be kept in a secure place at the clinical site.

### Masking Procedures

In this trial all personnel from QIMR and Q-Pharm will be blinded to the randomization scheme, except for the member(s) of the study team at Q-Pharm who are responsible for preparing and administration of the vaccine and saline as well as the statistician supporting the SMC (as necessary).

In a location in the trial clinic where blinded study personnel are not present, the unblinded study pharmacist will prepare that study product for injection and an unblinded nurse will check the dose and administer the product. To maintain the blind, the study participants will place their arms on which the vaccine will be administered under a curtain barrier, thus they will not know whether study product or control is being administered.

Documentation will be made in the study record indicating the date and time of study product administration. The participant, the study personnel who perform study assessments after product administration, investigators, data entry personnel at the sites, and laboratory personnel performing immunologic assays will also be blinded to treatment assignment.

The unblinded member of the team will be instructed not to share treatment code information with the participant, QIMR staff, or the Q-Pharm study team staff at the clinical site. In addition, the unblinded staff will not perform any other study related duties apart from the preparation and administration of the study product.

### Termination of Study

The study may be terminated before of after continuation with the vaccine second dose:

- If serious safety concerns are identified by the Clinical Investigator, Program Investigator, QIMR-HREC, the TGA sponsor QIMR or the appointed Safety Monitoring Committee.

- In the event that the TGA sponsor (QIMR) chooses to discontinue or terminate the study, appropriate notification will be given to the investigator.
- If the study is terminated, participants who have received the investigational product will be followed for safety assessments as described in the Section on Early Subject Discontinuation.

### Reasons for Withdrawal

A participant will not receive further study product if unexpected serious clinical adverse event (SAE), an illness or medical condition, or a situation occurs such that in opinion of the Clinical Investigator, continued receipt of vaccinations would not be in the best interest of that person. Participants may voluntarily withdraw from receipt of future product administration and/or follow-up at any time.

### Handling of Withdrawals

If the participant is withdrawn from the study procedures or follow-up for any reason, with the participant’s permission, medical care will be provided for any SAEs that might have had occurred during the individual participation in the study until the symptoms of any SAEs are resolved and the participant’s condition becomes stable. If withdrawal from further product administration occurs, if willing the participant will be asked to continue the scheduled evaluations and to complete the early termination evaluation as described in section 7.6.

# Study Intervention/Investigational Product

## Study Product Description

The antigen component in the proposed vaccine candidate is an acetylated synthetic peptide (AcJ8) conjugated to diphtheria toxoid (D) carrier molecule. The J8 peptide is synthesized on a solid phase resin purified using preparative HPLC, acetylated (AcJ8) and then conjugated to diphtheria toxoid (D) carrier molecule. The AcJ8-D conjugate is adsorbed onto alum (2% Aluminum hydroxide, Alhydrogel). The vaccine formulation also contains phosphate as a pH stabilizer in an isotonic 0.9% sodium chloride solution. The vaccine formulation is a sterile, white, homogeneous suspension intended for intramuscular injection that will be supplied in single dose vials.

### Acquisition

The vials containing the vaccine candidate will be stored at Q-Gen prior to initiation of the study.

### Formulation, Packaging, and Labeling

Each 0.5 mL dose of the vaccine candidate contains approximately 15 μg of AcJ8 peptide, 35 μg of diphtheria toxoid and between 315-715 μg elemental aluminum as determined by assay.

The vaccine will be aliquoted in clear type I glass vials. All vials will contain sufficient amount of formulated vaccine for a single dose of 0.5 mL volume. The vials containing the aliquoted vaccine formulation will be labeled in accordance to the GMP guidelines.

The saline control is a sterile 0.9% sodium chloride solution and it will be administered in the participants randomized to receive placebo. Saline will be sourced by Q-Pharm in 2 mL ampoules. Each vial will be utilized for a single dose.

### Product Storage and Stability

The vaccine candidate will be aliquoted in clear Type I glass vials in a total volume of 0.7mL ± 5%, which is approximately 40% overfill of the amount required for administration. This amount will be sufficient to aliquot a single dose injection of 0.5mL. All vials containing the aliquoted vaccine candidate will be stored at 5°C ± 3°C, protected from light in the provided packages.

The content in the vaccine candidate vials MUST NOT BE FROZEN. Any vials containing frozen vaccine contents must be discarded.

The saline vials will be handled and stored at room temperature as per package insert.

## Dosage, Preparation and Administration of Study Intervention/Investigational Product

**Study Intervention vaccine**

Preparation:

The vaccine formulation is ready-to-use suspension. The vaccine suspension must be mixed well by gently inverting the vial to resuspend any deposits that might form during the storage. After mixing, the vaccine formulation has a whitish color. Prior to administration the vaccine suspension must be examined visually. If any discoloration or particulate is observed, the vial should be discarded. The vaccine should be kept on cold until it is administered.

Administration:

Each vial contains a sufficient quantity of vaccine suspension for a single injection. 0.5mL of the vaccine suspension from each vial is removed for a single dose. The first dose will be administrated intramuscularly (I.M.) in the deltoid region of the non-dominant arm or the arm opposite that used for blood draw. The second dose will be administrated in the alternate arm.

Dosage:

Each study participant randomized to receive the treatment will receive two injections at 8 weeks intervals.

**Saline**

Preparation:

Each dose consists of ready-to-use sterile 0.9% sodium chloride solution. Before use, the saline vial will be inspected to verify the labeling, that the vial is intact and contains a clear liquid solution. The saline vial will be discarded if the contents of the vial appear otherwise. Each vial is for use in a single participant only, and any residue will be discarded.

Administration:

The saline control will be administrated as a single 0.5 mL I.M. injection in the deltoid region of the non-dominant arm or the arm opposite that used for blood draw. The second dose will be administrated in the alternate arm.

Dosage:

The proposed dosage of the saline control in this study is 2 injections at 0.5mL volume at 8 week intervals.

## Accountability Procedures

Q-Pharm will request shipment of the vaccine candidate from Q-Gen prior to the initiation of the study, when the approval has been obtained from the QIMR - HREC Ethics Committee. The vials containing the saline control will be supplied by Q-Pharm. Vials containing the vaccine candidate and the saline control will be inventoried prior to the beginning of study enrollment on study accountability logs in regards to condition upon receipt, vial quantities, formulation/type of study product, lot and vial numbers. The Clinical Investigator (CI) or qualified study person designated by the CI will ensure that the received study products are the specified formulation. All vials will be placed into a locked temperature monitored refrigerator (5°C ± 3°C) protected from light until completion of the trial. Each dose of study vaccine or the saline control used or wasted will be accounted for in writing on the study accountability log at the time of the removal of the product from storage.

The unblinded study staff member who will prepare the study product will be responsible for maintaining accurate records in all the study accountability logs, as well ensuring that the vaccine vials are used in accordance with the protocol. Study products and study accountability logs will be available to QIMR or QIMR’s representative as part of the study monitoring procedures.

At the completion of the study, a clinical monitor will review final accountability records and QIMR will provide instruction for the used vials and the unused study products. The vials will be returned to the TGA sponsor (QIMR) or will be destroyed according to the standard process at the clinical site.

## Concomitant Medications/Treatments

Concomitant medications/treatments will be recorded during the 0-7 days following each dose and at the scheduled study visits (see Appendix A) and collected surrounding adverse events/serious adverse events. This documentation will include any prescription or nonprescription drug or other treatment. The name of the medication, start and stop dates of treatment, total daily dose, route of administration and indication will be recorded on the source documents.

Permitted concomitant medications:

- These medications include licensed hormonal contraceptives, antihistamines and decongestants for seasonal allergies, non-steroidal anti-inflammatory drugs, (e.g., ibuprofen), non-narcotic analgesics (e.g., paracetamol/acetaminophen) and antibiotics as medically indicated.

At each of the scheduled visits circumstances that could potentially interfere with the vaccine-induced immunity will also be checked for. These circumstances include:

1. Use of any investigational drug or investigational vaccine other than the study vaccine during the study period.

2. Administration of licensed vaccines within 30 days prior to screening and enrollment through to 30 days following the last study vaccine administration, with the exception of licensed inactivated influenza vaccines.

3. Administration of immunosuppressants or other immune-modifying drugs (as listed in the exclusion criteria)

4. Administration of immunoglobulins and/or any blood products up to 28 days after the last dose of vaccine.

5. Antibiotics prescribed for streptococcal throat infection.

If any of the participants require medications/treatments that are listed as exclusion criteria, the participant will be excluded from receiving further doses of the vaccine and will not be included in the immunogenicity evaluations. However, the participant will be encouraged to continue the scheduled follow-up visits and remain in the study for safety evaluation for doses already received.

# Study Schedule

Each eligible participant will be requested to visit the clinical site at enrollment and at each subsequent scheduled visit in accordance to the Schedule of Procedures (Appendix A). The evaluations that will occur at each visit are listed below.

## Screening Period (Day –28 to Day –7)

Informed consent will be obtained from all potential participants prior to initiation of any screening. The screening will be a two part process involving 1-2 visits. This process will be carried out in accordance with the guidelines for Good Clinical Practice (ICH E6) [4]. The consent process will be initiated by the CI or an appropriately qualified study person designated by the CI. Detailed instructions for obtaining and documenting informed consent of study subjects are outlined in section 14.3 Informed Consent Process.

After informed consent has been obtained, study eligibility will be assessed for each prospective participant. A complete medical history will be obtained (Appendix E) as well as a review of medication history including concomitant medications. A physical examination including measurement of vital signs will be performed as well as cardiac auscultation by a physician. A number of screening tests will also be performed. Please refer to appendix D for a comprehensive list of tests to be performed at screening. These tests include:

• Serum β-HCG for all female participants;

• Routine hematology and biochemistry and urinalysis by dipstick test; (see Appendix D);

• Urine Drug Screen and alcohol breath test;

• Serum samples to be collected for ASOT, α-DNase B and α-J8 peptide antibodies;

• Throat swab and rapid antigen test for presence of Group A streptococcal infection

• Electrocardiogram (ECG) and echocardiogram (ECHO);

Participants who complete all screening procedures and satisfy all entry criteria will be considered eligible to participate in this study. To be eligible for study entry, clinical laboratory values at screening must not be clinically significant when outside the range of the normal values in the testing laboratory. Re-screening will not be allowed unless the CI considers the cause of the initial pre-screening failure to be of an acute and completely reversible nature.

If screening laboratory results are abnormal, e.g., HIV testing, the participant will be will be referred for appropriate counseling. If any clinically significant abnormalities are detected during screening, the participant will be referred for follow-up tests to a general practitioner or medical specialist as appropriate.

## Enrollment/Baseline

Participation consent must be obtained from all eligible participants prior to study product administration.

**Day 0, (Day of Dose 1)**

Two participants, one randomized to receive vaccine study product and the other control saline will have study product administered 24 hours prior to the remaining 18 participants which may be split into 2 cohorts. The safety of the first dose in these two participants will be initially observed for at least 6 hours and evaluated by the clinical investigator and the ISM to make recommendation on whether to proceed with administration of the study product to the remaining 18 participants.

These 2 participants will follow the events listed in Day 0 and will be dosed on Day 56 as all the participants of the study.

The safety of the first dose of the vaccine candidate in the study participants will be evaluated by a SMC that will make a recommendation to proceed to the administration of the next vaccine dose. See section 4 for the vaccine candidate dosage schedule. Refer to Appendix A for a tabular presentation of the study procedures.

Procedures to be followed at enrollment:

1. Review study eligibility and record concomitant medications.

2. Review medical history for any change in status from screening

3. Record baseline symptoms and vital signs. If the participant is febrile (temperature >38°C [>100.4F] or has an acute illness, the dose must be rescheduled. If rescheduled, the study product administration must be within 28 days after all screening procedures.

4. Targeted physical examination including cardiac auscultation. Particular attention to any elicited symptoms and signs consistent with rheumatic fever i.e. rash, arthalgia/arthritis (joint pain/swelling), renal angle tenderness and cardiovascular examination and movement disorder.

5. Obtain a throat swab and conduct a rapid antigen test for group A streptococci

6. Collect urine for urinalysis and blood for hematology, biochemistry and serology including, ASOT, α-DNase B, detection of α-J8 peptide vaccine specific antibodies, blood sample for quantitative cellular response as well as serum samples for α-DT antibodies and safety serum storage (refer to appendix D for tests required and blood volumes).

7. For female participants ensure that adequate contraceptive precautions have been taken since the screening pregnancy test and ensure that a urine test for β-HCG is negative.

8. Perform breath alcohol test and urine drug screen.

9. Administer the study product.

10. Observe the first two sentinels, the first two participants for at least 6 hours after study product administration to evaluate for any adverse reactions.

11. For all other participants, observe for at least 30 minutes after study product administration to evaluate for immediate adverse reactions.

12. Repeat vital signs after minimum of 30 minutes after study product administration or at least before discharge. Record any immediate injection site reaction or systemic reactions if they occur.

13. Educate the participant during the post-study product administration waiting period on proper use of the thermometers and injection site reaction measurement aid. Discuss the signs and symptoms of potential adverse events (AEs) and review the instructions on completion of the memory aid.

14. Distribute Day 0-7 Memory Aid (MA).

## Treatment Period

The activities performed during the treatment procedures will be initiated by the investigators of the study or a qualified study person designated by the CI. Please refer to the table Appendix A for the activities that will occur at each of the scheduled visits.

**Day 2 ± 1 day (2 days after Dose 1, telephone call)**

1. Enquire for any acute complaints/adverse events/serious adverse events.

2. Review MA for solicited local and systemic reactogenicity and unsolicited events.

3. Review concomitant medications.

**Day 8 ± 2 (8 days after Dose 1, telephone call)**

1. Enquire for any acute complaints/adverse events/serious adverse events.

2. Review MA for solicited local and systemic reactogenicity and unsolicited events

3. Review concomitant medications.

**Day 28 ± 7 (28 days after Dose 1)**

1. Review medical history for any change in status from last study visit

2. Review any acute complaints/adverse events/serious adverse events.

3. Review concomitant medications.

4. Record vital signs.

5. Targeted physical examination including cardiac auscultation. Particular attention to any elicited symptoms and signs consistent with rheumatic fever i.e. rash, arthalgia/arthritis (joint pain/swelling), renal angle tenderness and cardiovascular examination and movement disorder.

6. Collect urine for urinalysis and blood for hematology, biochemistry and serology including ASOT, α-DNase B, detection of J8 vaccine specific antibodies and cellular responses and quantative cellular response.

7. Perform ECG and ECHO.

**Day 56 ± 5 (Day of Dose 2)**

1. Ensure that an approval has been granted from SMC to proceed with the second study product administration.

2. Review inclusion/exclusion criteria.

3. Review medical history for any change in status from last study visit.

4. Review any acute complaints/adverse events/serious adverse events.

5. Review concomitant medications. If the participant has received an antibiotic treatment within 14 days prior to this administration, at discretion of the CI the participant may be withdrawn from the study or the administration of the second dose rescheduled within the allowed window for administration of the dose.

6. Record baseline symptoms and vital signs. If the participant is febrile (temperature >38°C [>100.4F] or has an acute illness, the study product administration must be rescheduled within the allowed window for administration of the dose.

7. Obtain a throat swab and conduct a rapid antigen test for group A streptococci.

8. Obtain a urine sample from all female participants for β-HCG testing. Ensure that the testing is negative before study product administration.

9. Perform breath alcohol test and urine drug screen.

10. Collect urine for urinalysis and blood for hematology, biochemistry and serology including, ASOT, α-DNase B and detection of α-J8 peptide vaccine specific antibodies and blood sample for quantitative cellular response as well as serum samples for α-DT antibodies and safety serum storage (refer to appendix D for tests required and blood volumes).

11. Targeted physical examination including cardiac auscultation. Particular attention to any elicited symptoms and signs consistent with rheumatic fever i.e. rash, arthalgia/arthritis (joint pain/swelling), renal angle tenderness and cardiovascular examination and movement disorder.

12. Administer the study product in the alternate arm from previous dose.

13. Observe for at least 30 minutes after study product administration to evaluate for immediate adverse reactions. Repeat vital signs after approximately of 30 minutes after study product administration, or at least prior to discharge. Record any immediate injection site reaction or systemic reactions if they occur.

14. Educate the participant during the post-study product administration waiting period on proper use of the thermometers and injection site reaction measurement aid. Discuss the signs and symptoms of potential adverse events (AEs).

15. Distribute Day 0-7 post second study product administration MA and review the instructions on completion of the memory aid.

**Day 58± 1 (2 days after Dose 2, telephone call)**

1. Enquire for any acute complaints/adverse events/serious adverse events.

2. Review MA for solicited local and systemic reactogenicity and unsolicited events

3. Review concomitant medications.

**Day 64 + 2 (8 days after Dose 2, telephone call)**

1. Enquire for any acute complaints/adverse events/serious adverse events.

2. Review MA for solicited local and systemic reactogenicity and unsolicited events

3. Review concomitant medications.

**Day 84 ± 7 (28 days after Dose 2)**

1. Review medical history for any change in status from last study visit

2. Review any acute complaints/adverse events/serious adverse events.

3. Review concomitant medications.

4. Record vital signs.

5. Collect urine for urinalysis and blood for hematology, biochemistry and serology including ASOT, α-DNAse B, detection of α-J8 peptide specific antibodies and quantitative cellular response.

6. Targeted physical examination including cardiac auscultation. Particular attention to any elicited symptoms and signs consistent with rheumatic fever i.e. rash, arthalgia/arthritis (joint pain/swelling), renal angle tenderness and cardiovascular examination and movement disorder

7. Perform ECG and ECHO.

## Follow-up

See Appendix A for a tabular presentation of study procedures at these visits.

**Day 180 ± 14 (approximately 17 weeks after the last injection)**

1. Review medical history for any change in status from last study visit

2. Review any acute complaints/adverse events/serious adverse events.

3. Review concomitant medications.

4. Record vital signs.

5. Targeted physical examination including cardiac auscultation, paying particular attention to any elicited symptoms and signs consistent with rheumatic fever (rash, arthalgia/arthritis, renal angle tenderness abnormal cardiovascular findings or movement disorder.

6. Collect blood for hematology, biochemistry and serology including ASOT, α-DNAase B, and detection of α-J8 peptide vaccine specific antibodies and quantative cellular response. Collect urine for urinalysis.

**Day 266 ± 14 (approximately 30 weeks after the last injection)**

1. Review medical history for any change in status from last study visit

2. Review any acute complaints/adverse events/serious adverse events.

3. Review concomitant medications.

4. Record vital signs.

5. Targeted physical examination including cardiac auscultation, paying particular attention to any elicited symptoms and signs consistent with rheumatic fever (rash, arthalgia/arthritis, renal angle tenderness abnormal cardiovascular findings or movement disorder.

6. Collect blood for hematology, biochemistry and serology including ASOT, α-DNAase B, and detection of α-J8 peptide vaccine specific antibodies and quantative cellular response. Collect urine for urinalysis.

## Final Study Visit

See Appendix A for a tabular presentation of study procedures at these visits.

**Day 350 ± 14 (approximately 42 weeks after the last injection)**

1. Review medical history for any change in status from last study visit

2. Review any acute complaints/adverse events/serious adverse events

3. Review concomitant medications.

4. Record vital signs.

5. Targeted physical examination including cardiac auscultation by a physician. Paying particular attention to any elicited symptoms and signs consistent with rheumatic fever (rash, arthalgia/arthritis, renal angle tenderness and abnormal cardiovascular findings or movement disorder).

6. Collect blood for hematology, biochemistry and serology including ASOT and α-DNAse B, detection of α-J8 peptide vaccine specific antibodies, blood sample for quantitative cellular response as well as serum samples for α-DT antibodies and safety serum storage (refer to appendix D for tests required and blood volumes). Collect urine for urinalysis.

7. Perform ECG and ECHO.

If a participant’s ECHO is abnormal on the last scheduled visit, evaluations will continue until the ECHO results are read as normal or the condition has stabilized.

At the completion of the study, a letter will be forwarded to all participants’ general practitioners (GP) to inform them of:

• The participation of their patients in this study and the type of vaccine that they have received.

• The titer of the antibody response to the vaccine antigen, including the anti diphtheria toxoid antibodies measured at the final study visit with recommendation for any further administration of diphtheria toxoid containing vaccines.

• Any ongoing associated and not associated AE

## Early Termination Visit

Each participant may withdraw voluntarily at any stage of the study for any reason. Participants who withdraw from receiving further study product will however be encouraged to continue follow up visits as per schedule for the purpose of monitoring for safety outcomes.

If voluntary withdrawal occurs at any stage of the study, and the participant does not wish to continue the follow up visits the participant will be asked to complete an end-of-study evaluation. Participation in an end-of-study evaluation by each participant is voluntary. Activities during early termination visit will include:

• Review of Concomitant Medications

• General physical examination, including the injection site examination, will be performed;

• Review of memory aid at the termination visit if it has not been done previously;

• A cardiac evaluation by cardiac auscultation, electrocardiogram and transthoracic echocardiogram

• Blood samples will be taken for hematology and serum/biochemistries analyses, serum for safety serum storage and antibody for ASOT, α-DNAse B, and ELISA for J8 and D vaccine specific antibodies; Urine samples will also be collected for urinalysis by dipstick.

• In a case of occurrence of serious adverse events, regardless of whether or not it is judged to be vaccine related, the participant will receive appropriate care under clinical supervision until all the symptoms of the serious adverse events have diminished or resolved and the participant’s condition improved.

For ongoing AEs care will be provided for a period of time as specified in the clinical site work instruction protocols. However, if the nature of the ongoing adverse event is determined by the CI not being vaccine associated the participant will be advised to visit his/her own GP for further clinical care that he/she might require.

## Unscheduled Visit

In the event that a participant has an unscheduled visit, the visit will be documented. Data collected would include information appropriate to the situation, such as event history, a limited physical examination and/or laboratory tests.

# Study Procedures/Evaluations

## Clinical Evaluations

Active surveillance by clinical personnel will be undertaken for each participant at enrollment prior to administration of the first injection and at all clinic visits.

• Clinical personnel involved in this study will interview each participant at screening to collect information regarding the medical history of all the participants. At screening, this will include demographic information, age and ethnic origin, personal and family history of all known diseases or conditions, especially those listed in the exclusion criteria. Information will also be collected whether the participant had received vaccination with a diphtheria toxoid containing vaccine within the previous 5 years (Diphtheria toxoid, tetanus toxoid, acellular pertussis vaccines, diphtheria toxoid and tetanus toxoid vaccines, pneumococcal conjugate vaccine, Menactra) as this vaccination is an exclusion criterion for participation in the study. Medical history will be reviewed prior to administration of each vaccine dose and at every clinic visit (see Appendix A).

• A medication history will also be collected at screening, during all study visits and surrounding any adverse/serious adverse events. It will include all medications that are currently taken by all participants regardless of whether they are prescription medications for treatment of persistent condition such as antidepressants, antibiotics, contraceptive medications (for females), other vaccines or over-the-counter medications.

• Vital signs will be collected and acute complaints examined by clinical personnel prior to each study product administration and at all scheduled clinic visits. Each participant will be subject to a physical examination by a physician at screening to evaluate the general health status of the participant. This will include body weight, vital signs (blood pressure, temperature, heart rate and respiratory rate), an examination of vital organs and cardiac auscultation. Targeted (abbreviated) physical examination including cardiac auscultation will also be performed prior to study product administration of each injection and at all scheduled study visits (Appendix A). Exam will include particular attention to any elicited symptoms and signs consistent with any adverse events. Any deviations from normal will be reported to the study physician for a review. All deviations from normal detected by physical examination will be graded as 1 (mild), 2 (moderate), 3 (severe) and 4 (life threatening) (see Appendix B).

• Transthoracic echocardiography (ECHO) and electrocardiogram (ECG) tests will be performed for each participant in this study at screening, 28 days after each injection and at the final visit on day 350 by a technician blinded to the type of treatment that the participants are receiving. A single echo technician and a single cardiologist have been recruited to perform and interpret all ECHO findings. The technician will be located at the clinical site and the cardiologist reading the findings will be located in the hospital which is physically separated from the clinical site where study activities will be conducted. There will be no discussion regarding the clinical trial between the trial personnel and the hospital personnel. Echocardiograms will be reviewed for any abnormalities by the study cardiologist who will be also blinded to type of treatment that the participants are receiving. If any findings are identified as abnormal a second cardiologist will be asked to independently review the test results and report on the findings. If an abnormal ECHO/ECG is confirmed, it will be reported to the Independent Safety Monitor and Safety Monitoring Committee to determine whether it meets halting rules. Any change in degree of mitral regurgitation with EROA 10-19mm2 but a change of <10mm2 from baseline will be followed on a monthly basis with echocardiogram on a monthly basis until stable (Appendix H).

• The injection site will be examined immediately before and after each dose. The reactogenicity of the study product will be determined by assessing the local pain, tenderness and pruritis and measuring the erythema/redness and induration/swelling at the injection site using the injection site reaction measurement tool (Appendix C).

• Solicited systemic symptoms (see section 9.2.1) will be assessed prior to study product administration and at 30 minutes after each dose. Memory aids will be given to each participant on each product administration day. All participants will be asked to record the occurrence of any injection site reaction or general symptoms for 7 days following each dose in memory aids as indicated in section 9.2.1. A telephone call will be made by a study nurse to each participant at 2 days and 8 days after each injection. All participants will be encouraged to call the clinical personnel involved in this study on the provided contact details, or to visit the clinical site if they observe any reactions, grade 3 or above as specified in this protocol (see Appendix B), or if they have any concern. Any AE that occurs greater than 7 days after each dose should be reported to the clinical study personnel during the scheduled interim visits or by calling the designated contact person. All AEs will be assessed during each study visit.

## Laboratory Evaluations

### Clinical Laboratory Evaluations

The collection of blood and urine samples for analysis will be carried at the clinical site (Q-Pharm, Australia). Analyses for the biochemical hematological results including the serum pregnancy test and the microscopic urine analysis (if required) will be performed at a nominated pathology laboratory. Tests performed at Q-Pharm include, urinalysis, urine pregnancy test, alcohol breath test, urine drug test and rapid antigen test for group A streptococci. It will be performed with QuickVue In-Line Strep A test which is currently not licensed in Australia, however it is licensed and widely used in US. These tests will be imported from US for the purpose of use in this trial only. The test will be used as per manufacturer’s instructions. Laboratory evaluation will be undertaken for all participants at screening, prior to each immunization and at every scheduled clinic visit, (refer to Appendix D for the tests required and blood volume for different samples). The samples required include:

• Blood sample for hematology, serum biochemistry and serology including ASOT and α-DNAse B (please refer to Appendix D for samples types, volumes and tests that will be undertaken at different time point including the laboratories that will perform the tests)

• Urine sample for dipstick urinalysis for all participants and for pregnancy test for female participants.

The laboratory parameters to be monitored are as follows:

• Hematology: full blood count (FBC) [hemoglobin (Hb), red blood count (RBC), hematocrit (Hct), mean cell volume (MCV), platelet count (Plts)], total white blood cell count (WBC) and differential,

• Serum/Biochemistry: Sodium, Potassium, Glucose, Bicarbonate, Chloride, Urea (Blood Urea Nitrogen; BUN), Creatinine, Calcium, Phosphorus, Creatinine Albumin, Total Bilirubin, At screening parameters to be measured will include: Creatine Phosphokinase (CPK), Sodium, Potassium, Bicarbonate, Chloride, Liver Function tests (LFTs: Alkaline Phosphatase, Alanine Aminotrasferase (ALT), Aspartate Aminotrasferase (AST)),

• Cardiac troponin T, C-reactive protein (CRP), C3 complement, Anti-streptolysin O Titer (ASOT) and α-DNAse B antibody levels, auto-antibody screen (ANA) and Anti-CCP antibody,

• A β-HCG serum pregnancy test will be performed for all female participants at screening. Urine pregnancy tests will be performed prior to each dose. The results must be available and negative prior to each administration of study product.

• As part of the screening process, blood samples from the participants will be tested for antibodies for blood borne viral pathogens including Human Immunodeficiency virus, type 1 and type 2 (HIV-1/-2), Hepatitis C virus (HCV), Hepatitis B virus surface antigen (HBsAg).

• Urinalysis: urinalysis including protein, glucose and hemoglobin; Complete urinalysis to be performed with microscopic analysis if the dipstick urinalysis is abnormal (any blood or > trace protein).

• Urine Drug Test: At screening, and on pre vaccine administration (Day 0 and Day 56). The urine drug screen can detect the drugs such as Amphetamines, Methamphetamines, Barbiturates, Benzodiazepines, Cocaine, Methadone, Opiates, Phencyclidine, Tetrahydrocannabinols and Tricyclic antidepressants at the Q-Pharm clinic. If the result of the test is positive volunteers may be allowed to continue, or may be delayed or withdrawn according to site specific instructions. This will also include an alcohol breath test at screening, on Day 0 and day 56 prior to vaccine administration.

### Special Assays or Procedures

Serum samples will be required for evaluation of the serologic responses of the vaccine candidate, including ELISA and bactericidal abilities of the vaccine induced J8 specific antibodies as well as antibodies to diphtheria toxoid.

Sera samples will also be kept for each participant as indicated in this protocol and tested should better assay become available for detecting cross reactive antibodies that have been induced due to the vaccine candidate.

Samples of sera will be collected from the participants prior to each injection, at day 28 after each injection and at the scheduled clinic visits as indicated in this protocol. Immunogenicity to vaccine components will be measured by ELISA and indirect bactericidal assays. Sera will be stored and assays will be conducted on batched samples after all sera have been collected.

• Standard ELISA will be performed to determine the level of J8 peptide-specific serum IgG concentrations (µg/ml) in sera of all participants at the time intervals described in Appendix A. Purified human peptide specific antibodies from sera obtained from donors will be used to generate a standard curve, as well as a positive control for each ELISA plate.

• Indirect Bactericidal Assay: This assay will be used to measure the neutralization abilities of the vaccine-induced antibodies against different standard group A streptococcus strains in accordance to the protocol specific SOP. In this assay the percent reduction on GAS colony forming units (CFU) will be determined. As a control a panel of donors will be used to determine the base level of bacterial growth.

• The change in the baseline level of anti-D antibodies in the sera of all participants will be investigated at the end of the study. Anti-D antibodies will be measured by ELISA.

### Specimen Preparation, Handling, and Shipping

#### Instructions for Specimen Preparation, Handling, and Storage

All samples will be collected in accordance to the nominated laboratory procedures and tested in accordance to approved SOP for the specified testing. Collected sera samples will be batched and stored at -20 °C or below until required for testing unless otherwise specified.

Participants will be asked to provide consent for future use of the retained samples in the consent form for research related to group A streptococcus vaccine development. Once this study is completed, samples belonging to participants who do not consent to have their samples to be used in future research will have all labels removed and the samples will be destroyed. Samples from participants who agree for their samples to be used for future research use will remain stored at -80 °C. Before future use of these samples, the project intended for use will undergo ethical and scientific review by the appropriate ethics committee.

# Assessment of Safety

## Specification of Safety Parameters

Adverse events will be monitored starting with the observation period post product administration through the duration of the study.

## Methods and Timing for Assessing, Recording, and Analyzing Safety Parameters

### Adverse Events

The CI is responsible for reporting all AEs that are observed or reported during the study, regardless of their relationship to the study product administered.

According to ICH E6 Good Clinical Practice Guidelines [3], an Adverse Event (AE) is defined as any untoward medical occurrence in a participant regardless of its causal relationship to the study treatment. An AE can therefore be any unfavorable and unintended sign (including an abnormal laboratory finding), symptom, or disease temporally associated with the use of medicinal (investigational) product. Thus, any change from the baseline would be referred as an adverse event.

Unsolicited adverse events will be captured during observation and examination of each participant through the course of the study and recorded using study source documents. All participants will be required to record any observed adverse event regardless of the relationship to the study product in the provided memory aid if it occurs within 7 days after the administration of the study product. Any AE that occurs after the memory aids have been reviewed should be reported to the clinical study personnel during the scheduled interim visits and interviews or by calling the designated contact person.

Each solicited adverse event will be collected from reviewing the memory aids through interview with the participant or on the source document designed to collect solicited reactogenicity. Solicited adverse events will be graded for intensity, according to the criteria in the table below. Solicited adverse events include the following reactions:

• Local reactions:

Redness / erythema

Swelling / induration

Pain at the injection site

Tenderness

Itchiness (pruritus)

• Systemic reactions:

Fever

Malaise (feeling unwell),

Diarrhea

Loss of appetite (anorexia)

Vomiting

Headache

Myalagia (muscle ache)

Joint pain/swelling (arthalgia/arthritis)

Tiredness

Rash

Nausea

Feeling feverish

Chills

Shortness of breath (dyspnoea)

Wheezing

Abdominal pain

The solicited injection site reactions, such as redness/erythema and swelling/induration are to be measured with the provided injection site reaction measurement tool (Appendix C). For the pain at the injection site and the systemic symptoms, the following guidelines will be used to quantify intensity see below and Appendix B:

| **Local reaction to study product** | **Mild**  **(Grade 1)** | **Moderate (Grade 2)** | **Severe**  **(Grade 3)** | **Potentially Life Threatening (Grade 4)** |
| --- | --- | --- | --- | --- |
| Pain | Does not interfere with usual activities | Repeated use of non narcotic pain reliever  > 24 hours or interferes with activity | Any use of narcotic pain reliever or prevents daily activity | Emergency room (ER) visit or hospitalization |
| Tenderness | Mild discomfort to touch | Discomfort with movement | Significant discomfort at rest | ER visit or hospitalization |
| Erythema/  Redness | 2.5 - 5 cm | 5.1 – 10 cm | > 10 cm | Necrosis or exfoliative dermatitis |
| Induration/Swelling | 2.5 - 5 cm and does not interfere with activity | 5.1 – 10 cm or interferes with activity | > 10 cm or prevents daily activity | Necrosis |

| Systemic reaction to injectable product | **Mild**  **(Grade 1)** | **Moderate (Grade 2)** | **Severe**  **(Grade 3)** | **Potentially Life Threatening (Grade 4)** |
| --- | --- | --- | --- | --- |
| Fever (°C)^  (°F) | 38.0 – 38.4  100.4 – 101.1 | 38.5 – 38.9  101.2 – 102.0 | 39.0 – 40  102.1 - 104 | > 40  > 104 |
| Other solicited events | Does not interfere with usual activities | Repeated use of non narcotic pain reliever  > 24 hours or interferes with activity | Any use of non-narcotic pain reliever or prevents daily activity | Emergency room (ER) visit or hospitalization |
| Unsolicited events | Should be graded according to the criteria in Appendix B | | | |

Adverse events characterized as intermittent require documentation of onset and duration of each episode.

All unsolicited AEs must be graded for severity and relationship to study product (see below and Appendix B).

Information collected will include the type of the AE, the onset and the severity of the AE (see table above and appendix B). All changes in the severity of an AE should also be recorded on source documents or medical records to document the duration of the AE at each level of intensity. Adverse events characterized as intermittent require documentation of onset and duration of each episode.

If the CI has any doubt as to whether a clinical observation is an AE, the event will be recorded in the appropriate CRF.

The clinician’s assessment of the relationship of an AE to study product is part of the documentation process, but it is not a factor in determining what is or is not reported in the study. If there is any doubt as to whether a clinical observation is an AE, the event should be reported. All AEs must have their relationship to study product assessed using the following terms: associated or not associated. In a clinical trial, the study product must always be suspect. To help assess, the following guidelines are used:

• Associated – The event is temporally related to the administration of the study product and no other etiology explains the event.

• Not Associated - The event is temporally independent of study product and/or the event appears to be explained by another etiology.

Any medical condition that is present at the time that the participant is screened and found eligible should be recorded and be considered as baseline. However, if it deteriorates at any time during the study, it should be recorded as an AE.

### Adverse Event Severity – Definition

The severity of adverse events will be graded on a three point scale:

**Mild**: discomfort noted, but no disruption to normal daily activities.

**Moderate**: discomfort sufficient to reduce or affect normal daily activities.

**Severe**: inability to work or perform normal daily activities.

**Potentially Life Threatening:** participant at immediate risk of death

### Serious Adverse Events

According to the ICH E6 Good Clinical Practice Guidelines [3], a Serious Adverse Event (SAE) is defined as a medical event that occurs at any dose of the investigational product and results in any of the following:

• Death

• Life-threatening adverse drug experience (participant at immediate risk of death)

• Requires prolonged inpatient hospitalization

• Results in persistent or significant disability/incapacity

• Results in congenital anomaly or birth defect

• Other medical events that do not result in death, are life-threatening, or require hospitalization may also be listed as a SAE when, based upon appropriate medical judgment, they may jeopardize the participant and may require medical and surgical intervention to prevent one of the outcomes listed in this definition. Examples of such medical events include allergic bronchospasm requiring intensive treatment in an emergency room or at home, blood dyscrasias or convulsions that do not result in inpatient hospitalization, or the development of drug dependency or drug abuse.

If any AE is classified as a serious adverse event by the CI or the co-investigator, or if it meets the criteria for a SAE, the occurrence must be:

• recorded on the appropriate serious adverse event case report form, source documents i.e. medical records and SAE report forms for vaccines;

• followed through resolution by a study physician;

• reviewed by a study physician.

### Procedures to be Followed in the Event of Abnormal Laboratory Test Values or Abnormal Clinical Findings

Safety laboratory tests will be performed at the follow-up visits scheduled at 28 days after each vaccination (days 28 and 84) and days 180, 266 and 350. See section 8.2.1 for list of safety laboratory tests. Toxicity tables are provided in Appendix B of this protocol and grade the severity of the laboratory abnormality as mild, moderate or severe. Laboratory abnormalities will be assessed as associated or not associated as described in section 9.2.1.

## Reporting Procedures

The CI is responsible for reporting all AEs that are observed or reported during the study, regardless of their relationship to the study product. Safety reporting regulations are defined in the ICH GCP.

### Serious Adverse Events

All Serious Adverse Events as defined in section 9.2.2 above will be reported immediately to QIMR as the TGA sponsor as per relevant QIMR standard operating procedures.

Within 24 hours the CI or the study co-investigator becoming aware of an SAE should:

- - Notify the QIMR Regulatory Affairs and the independent medical monitor of the SAE occurrence
  - Complete the initial SAE report by completing the details on the QIMR SAE Report
  - Fax the completed initial SAE report to the:

**QIMR Regulatory Affairs Office**

**Fax No. 07 33620109**

The original signed copy should be mailed to:

**QIMR Regulatory Affairs Office,**

**Level 5, QIMR Central,**

**300 Herston Road, Herston Qld 4006**

- - The SAE report should be submitted to the QIMR-HREC by the QIMR PI via the E-Form system.

Within 14 days of onset of the SAE –

- The PI will complete the follow-up SAE report by filling in SAE follow-up details on the QIMR SAE Report and entering “Follow-up” on the “Report Type” field. The reporting investigator should sign and date the report.
- The completed Follow-up SAE report should be faxed to the:

**QIMR Regulatory Affairs Office**

**Fax No. 07 33620109.**

The original signed copy should be mailed to

**QIMR Regulatory Affairs Office,**

**Level 5, QIMR Central,**

**300 Herston Road, Herston Qld 4006.**

- The SAE report should be submitted to the QIMR-HREC by the QIMR PI via the E-Form system.

Other supporting documents of the event may be requested by the QIMR Regulatory Affairs or the ISM and will be provided as soon as possible.

Summary reports for the occurrence and the follow up of all SAE observed will be sent to the ISM on a monthly basis for a review and be incorporated into a report for SMC review. Once the reports have been reviewed by the ISM, copies from the reports will be faxed back to the clinical site to be filed with the source documents and the medical records.

### Regulatory Requirements

Events both serious and unexpected that are associated with the administration of the vaccines will be reported by the TGA sponsor (QIMR) or designees to the TGA regulatory authorities.

The reviewing and reporting to TGA of all serious and unexpected adverse reactions will comply with relevant QIMR policy and TGA requirements as outlined in the “Therapeutic Goods Administration (TGA), Access to Unapproved Therapeutic Goods - Clinical Trials in Australia” document and ”The Australian Clinical Trial Handbook”.

Following the notification from the CI or the co-investigator, the sponsor for TGA (QIMR), will notify TGA for occurrence of SAE within the required timelines:

• Fatal or life-threatening events will be reported to TGA within 7 calendar days with a follow up report to be submitted within 8 additional calendar days.

• A full report for all other serious and unexpected events should be submitted no later than 15 calendar days after first knowledge by the sponsor.

• All other reactions and adverse events will be reported to the relevant ethics committees and TGA at least annually in a summary format.

The Independent Safety Monitor will have direct contact with the CI who is responsible for QIMR reporting to the TGA in accordance with relevant QIMR policy.

### Reporting of Pregnancy

The occurrence of pregnancy at any stage during the trial will be recorded in the case report form. Any further administration of the study product will be discontinued. With the participant’s permission, she will be followed up in accordance to the clinical site work instructions for follow up pregnancies. The participant will be asked to continue the scheduled follow up visits until the following dose (if applicable) for safety evaluation for doses already received and then at the end of the study. Additionally, the pregnancy outcome will be recorded in the CRF.

## Type and Duration of Follow-up of Subjects after Adverse Events

All AEs will be followed up until resolved or stabilized. The medical care provided will be decided by the CI and clinical personnel involved in the study to provide the best outcome and recovery for the study participant. In general, all AEs will be recorded at each of the scheduled interim visits until AE are resolved or stabilized.

## Halting Rules

Study product administration in the trial will be halted for expedited ISM/SMC review if, during the 7 days after product administration, any of the following occur:

• One (1) or more participants experiences an SAE that is unexpected and associated with study product administration

• 10% or more of the participants enrolled to date experience a severe (grade 3) study product associated fever

• 10% or more of the participants enrolled to date experience a severe (grade 3) study product associated local redness or induration;

• 10% or more of the participants enrolled to date experience a severe (grade 3) study product associated subjective reaction (pain, headache, malaise or myalgia), the severity of which is corroborated by study personnel

• One (1) or more participants experience a study product -associated bronchospasm or other anaphylactic response

• One (1) or more participants experience a study product-associated allergic event or vaccine site ulceration, abscesses or necrosis

The trial will be halted at any time if there is:

• Any patterns of laboratory values or clinical symptoms that the CI considers to be a significant safety issue for participants or any clinical symptoms of rheumatic carditis and/or rheumatic fever as defined by Jones criteria. (see Appendix G).

• ECG or ECHO abnormality such as:

- Increase in valvular regurgitation to greater than mild severity;
- Development of mitral regurgitation with an EROA of ≥20mm^2^ or >10mm^2^ increase from the baseline study;
- An absolute increase in effective regurgitant area of ≥10mm^2^ compared to baseline;
- Development of mitral regurgitation with an EROA > 20 mm^2^ and

echocardiographic criteria of probable RHD will require notification of the appropriate regulatory bodies as associated SAE.

- Any abnormal valve leaflet thickening (subjective);
- Worsening left ventricular systolic function (ejection fraction of < 50%);
- Left ventricular dilatation (based on LV diameter > upper limit of reference ranges for LV end diastolic diameter (LVEDd) indexed for body surface area (Females 24-32 mm/sqm; Males 22-31mm/sqm);
- Significant right ventricular dysfunction;
- Evidence of pulmonary hypertension (estimated RVSP > 40mmHg);
- Development of more than trivial pericardial effusion;

Subsequent reviews of serious, unexpected and related AEs by the ISM, SMC, QIMR-HREC, QIMR or TGA may also result in suspension of further study product administrations. On initiative by the TGA and the sponsors, suspension may be applied for any additional enrollment or administration of the investigational vaccine for the entire study.

## Safety Oversight (ISM plus SMC)

The Safety Oversight in this study will be under the direction of both Independent Safety Monitor (ISM) and the Safety Monitoring Committee (SMC).

Independent Safety Monitor

The ISM is a physician with relevant expertise, whose primary responsibility is to provide independent safety monitoring in a timely fashion. Participation is for the duration of the study and the ISM is appointed by the sponsor. The ISM should be able to access participant records readily in real time. The primary focus of the ISM is to independently review serious adverse events that may be associated with product and adverse events of special interest. The ISM should investigate those events considered serious and unexpected. Clinical and laboratory data, clinical records and other study-related records should be made available for ISM review. It is the responsibility of the CI to ensure that that the ISM is appraised of all new safety information relevant to the study product and the study. This includes providing the ISM with a copy of the Clinical Investigator’s Brochure (CIB) in advance, as well as promptly providing all CIB revisions and all other safety information issued by the sponsor that is received by the CI. The ISM should receive all protocol revisions and may receive other documents related to the study.

The ISM will be in communication with the CI at the enrollment site and the SMC chair for any event that needs further evaluation.

The ISM together with the clinical investigator (CI) will be also involved in the review of all reactions including the solicited events that have occurred within the observation window for the two participants that will initially receive the study product. The ISM will also be involved in the decision making by SMC on whether the remaining participants will receive the first dose.

The ISM will not be directly involved with the trial and is not under the CI’s supervision and has no financial, intellectual, proprietary or professional interest in outcome of the trial.

Safety Monitoring Committee

The SMC will be composed of the ISM, an infectious diseases physician, a physician with expertise in clinical trials or vaccine studies and a cardiologist. The safety of each dose of the vaccine candidate through Day 28 in the 20 participants will be evaluated by the SMC following a review of a clinical and laboratory safety data package. The SMC will also review the recorded AEs and SAEs. The SMC makes recommendations to the sponsor. These recommendations are approved by the SMC chair who signs a letter of recommendation that is sent to the CI.

In regards to the safety review of the first dose, the SMC will provide recommendations to the sponsor for administration of the second dose. These recommendations will be communicated to the CI, PI and QIMR-HREC prior to administration of the second dose. There will also be a safety review by the SMC after the second dose with recommendations regarding the continuation or modification of the study.

Additionally, the SMC will meet to assess any events that trigger the halting rules or as needed to provide a recommendation and findings to QIMR-HREC, the PI and the CI, in accordance to the approved SMC charter.

Whether at a scheduled or unscheduled meeting, the SMC will consider safety signals to determine whether or not they can recommend that the study continue.

# Clinical Monitoring

The sponsor is responsible to ensure that this clinical study is monitored adequately and that it is being conducted in accordance to this protocol and regulatory requirements.

The monitoring visits will be in accordance to the current QIMR standard operating procedures and the outsourced CROs.

## Site Monitoring Plan

Site monitoring is conducted to ensure the human subject protection, study procedures, laboratory, study intervention administration, and data collection processes are of high quality and meet sponsor, GCP/ICH and, when appropriate, regulatory guidelines. Site visits may be conducted by an authorized representative of QIMR or other regulatory agencies to inspect study data, participants’ medical records, and CRFs in accordance with ICH guidelines, GCP and the respective local and national government regulations and guidelines.

The investigator will permit authorized representatives of QIMR and the respective local and national health authorities to inspect facilities and records relevant to this study if needed.

The monitoring visits will be conducted as specified in the site monitoring plan. Briefly, the monitoring visits will be conducted before, during and after the trial. However, the sponsor for TGA QIMR have authority to request initiation of additional monitoring visits.

In QIMR-sponsored trials, all activities associated with site initiation and monitoring will be outsourced to a Contract Research Organization (CRO). The monitor will visit the CI at the site of investigation to ensure that all requirements are being met as specified in this protocol. The CI will ensure that all follow up actions are sufficiently addressed and/or completed and appropriately documented within the recommended timeframe.

# Statistical Considerations

## Introduction

Group A Streptococcus (GAS) is a serious human pathogen that affects people of all ages and socioeconomic backgrounds. Common GAS infections include streptococcal pharyngitis and pyoderma, which are particularly common in children. Far more serious infections of GAS include necrotizing fasciitis, pneumonia, and toxic shock syndrome, primarily affecting the elderly. Post-streptococcal sequelae include rheumatic fever, rheumatic heart disease, and acute glomerulonephritis.

## Overview and Study Objectives

This study is a randomized, double-blinded, controlled Phase I clinical trial to evaluate the safety and immunogenicity of a Group A Streptococcus Vaccine in a novel J8-D/alum formulation performed in healthy adult participants from Brisbane, Australia. Twenty participants will be randomized in a 3:1 ratio to receive either two injections each containing 50 μg of the vaccine candidate or two injections of saline at eight-week intervals.

### Primary Outcome Measures

The primary objectives of this study are to:

• Evaluate the safety of a Group A Streptococcus vaccine candidate delivered intramuscularly to healthy adults in two doses of 50 μg AcJ8-D adsorbed onto alum.

Safety of the vaccine candidate will be assessed on the basis of the frequency and occurrence of injection site and systemic reactions following administration of the investigational vaccine. Safety parameters include changes in clinical biochemistry and hematology values on blood and urine, and changes in electrocardiogram and transthoracic echocardiograms. Adverse events and serious adverse events will be reported throughout the duration of the study.

### Secondary Outcome Measures

The secondary objective of this study is to assess the immunogenicity of a group A streptococcus vaccine candidate delivered intramuscularly in two doses of 50 µg AcJ8-D adsorbed onto alum.

The immune response to the AcJ8-D vaccine will be assessed by:

• Monitoring for the presence of vaccine-specific antibodies in all participants of the study and the titer of the vaccine-specific antibodies after each administered dose of the vaccine candidate, to investigate the relationship between the doses administered and the titer of vaccine-specific antibodies.

• Measuring the bactericidal activity of the induced antibodies against different standard reference strains of Group A Streptococcus such as M1, M6, and 88/30.

## Study Population

This study will be conducted in twenty healthy adults, both male and female, between the ages of 18-45 years, inclusive. Participants will be from the Brisbane, Australia area. Additionally, participants must have normal electrocardiogram and echocardiogram results and have no personal or family history of post-streptococcal or collagen-vascular disease.

## Study Design

This study is a double blind, randomized Phase I single cohort clinical trial with N=20 participants. The participants will be randomized 3:1 to receive either the investigational vaccine (N=15) or the control (saline, N=5. Each participant will receive a total of two injections at eight-week intervals.

Screening is expected to take three weeks, starting at Day -28. At Day 0, two sentinel participants will be enrolled and randomly selected to receive either the investigational vaccine or the control saline 24 hours before all other participants. Assuming that no immediate serious adverse reactions are observed in these sentinel participants, the remaining 18 participants will be enrolled and dosed. The sentinel participants will receive their second dose at the same time as the remaining participants.

Each participant will receive two injections administered at eight-week intervals with a safety review following each injection. The SMC review will include data on:

- Reactogenicity at one-week post-study product administration;
- Hematology, chemistry, and urinalysis at four weeks post-study product administration, and
- ECG and ECHO at four weeks post-study product administration.

Data will be collected for review at five weeks post-initial dose with an SMC review in the seventh week. There will be at least seven days between the SMC review and the next injection to permit adequate review of post-product administration safety data.

## Study Outcome Measures

The primary safety assessments will be based on the following outcomes:

• Solicited reactogenicity data acquired from a memory aid that will be kept for Days 0-7 following each product administration by each study participant.

- Local reactions following product administration include erythema (redness), induration (swelling), and pain, tenderness, and/or pruritis (itchiness) at the injection site.
- Systemic reactions following vaccination include feverishness, chills, diarrhea, anorexia (loss of apetite), vomiting, headache, malaise (feeling unwell), myalgia (muscle ache), nausea, rash, arthalgia/arthritis (joint pain/swelling), dyspnea (shortness of breath), wheezing, and abdominal pain.

• Participants will record their oral temperatures daily, as well as any changes that may suggest haematuria or cutaneous abnormalities

• Vital signs and a physical exam including organs targeted in rheumatic fever such as skin, joints, kidney and including cardiac auscultation will be acquired at each study visit.

• Clinical laboratory data acquired at four weeks post-study administration which include haematology, biochemistry, and urinalysis.

The secondary immunogenicity outcomes are based on the following outcomes:

• Production of peptide specific antibodies and increase in the titer for the specific antibodies from the baseline titer after product administration.

- The relationship between the doses administered and the titer of vaccine-specific antibodies

• The percent reduction in a bactericidal assay of group A streptococcus colony forming units (CFU) that elicited vaccine specific antibodies.

- The percent reduction in a bactericidal assay of group A streptococcus CFU achieved in M1, M6 and 88/30 (Australian clinical isolate) group A streptococcus strains

## Study Hypotheses

The hypothesis of this study is that the administration of the vaccine candidate will be safe and immunogenic. This study is not designed to conduct formal hypothesis testing. Only descriptive statistics will be used.

## Sample Size Consideration

This study is a Phase I safety and immunogenicity single cohort clinical trial consisting of N=15 investigational vaccine recipients and N=5 control saline recipients. Saline recipients are included to blind clinical and laboratory assessments. They may also serve as the basis of comparison should any study product safety concerns be detected. The analysis will be primarily descriptive, and will include tabular and graphical summaries of the safety and immunogenicity data, supplemented by a complete listing of outcomes for each participant. The sample size chosen will not provide sufficient power to test for differences or similarities amongst the study product urine and recipients. Preference will be given to reporting point and confidence interval estimates, rather than p-values associated with formal hypothesis testing. All analyses will be regarded as exploratory, and any significant findings will be regarded as hypothesis generating, rather than hypothesis confirming.

## Participant Enrollment and Follow-Up

Eligible participants will be enrolled from Brisbane, Australia. A follow-up visit will be conducted at Day 180 ± 14 and Day 266 ± 14, which is approximately 12 and 30 weeks respectively following the last injection). The final study visit takes place at day 350 ± 14 (~42 weeks after the last injection). At these visits, vital signs, blood will be collected and targeted physical examinations including cardiac auscultation will be performed. Additionally, the final study visit includes an ECG and ECHO.

The general practitioners (GPs) nominated by the enrolled participants will be informed by a letter that the participants are enrolled in this study and that they may have received a diphtheria toxoid-containing investigational study product. At the completion of the study, another letter will be sent to the participant’s general practitioners describing their participation in the study, including which study product they received, the titer of antibodies for both the vaccine antigen as well as the anti-diphtheria toxoid as measured at the last visit, and any ongoing associated and unassociated adverse events.

## Planned Interim Analyses

### Safety Review

Approximately seven weeks after the first product administration, the Safety Monitoring Committee (SMC) will review study data accumulated up to the five-week time point. The SMC is responsible for monitoring participant safety in this QIMR sponsored study. The SMC will review factors that might compromise the study, such as protocol deviations, and losses to follow-up, and the accumulated safety data.

The data presented to the SMC will not be separated by treatment group. Specifically, data from saline and vaccine candidate recipients will be pooled. SMC members may request additional unblinded analyses at their discretion. The SMC will make recommendations to the Chief Investigator and QIMR as to whether the study should continue, be modified, or terminated, and specifically, whether corrective actions are required, or the study may proceed as planned to the second dose of product.

In the SMC report, the demographics of study participants: age, gender, race and ethnicity; will be tabulated. A table of study completion status will display the counts of participants who are in follow-up, have terminated, or have completed the trial by study group. All subjects dropped from the study will be listed, with the date of termination, length of time on study, and reason for termination. Participant specific protocol deviations will be tabulated according to category and reason for deviation.

Reported safety data will include unsolicited adverse event reports, reactogenicity solicited at one-week post-product administration; hematology, chemistry, and urinalysis at four weeks post-product administration. Additionally, analyses will be conducted with ECG, ECHO at four weeks post-product administration and at the final study visit.

Adverse events will be classified and counts will be expressed in terms of the number and percentage of participants experiencing the event.

Local and systemic reactogenicity will be classified by reaction type, and the maximum grade over the follow-up period for each participant will be reported. The frequency of occurrence and severity of reactogenicity assessments will be summarized in tables and bar-charts.

Summary statistics for clinical laboratory results and vital signs will be calculated by dose number (1 or 2) and time point post-product administration, and will be displayed in both tabular and graphical form. Scatter plots will be used to display individual results by time-point, annotated by summary statistics and laboratory reference ranges. All individual abnormal results will be listed, along with a narrative interpreting the result, if available.

### Immunogenicity Analysis

No interim analyses of immunogenicity are planned.

## Final Analysis Plan

### Immunogenicity Analysis

The ELISA and Indirect Bactericidal Assays will be used to assess the immunogenicity of the vaccine candidate. The standard ELISA will be performed to determine the peptide-specific serum IgG concentrations (μg/mL) in sera of all participants at screening, and at Day 28 after each product administration as well as at days 180 ± 14, 266 ± 14 and 350 ± 14. The Indirect Bactericidal Assay will be used to measure the neutralization abilities of the vaccine-induced antibodies against different standard GAS strains. The percent reduction on GAS colony forming units will be determined.

No interim analyses of immunogenicity are planned. Assay results will be batched and analyzed at the end of the trial. Immunogenicity data will be collected pre-product administration and after each successive dose. All time points will be analyzed, but the focus will be on the immune response at 28 days after the final dose. Parameterizations of immune response will include a) the proportion of participants with a positive response (if criteria for a positive response are developed), b) the mean (or geometric mean) level of the response, c) the change in mean level (or fold-rise) from baseline, and d) the change in mean level (or fold-rise) between successive doses.

A detailed description of the final analyses will be developed in the statistical analysis plan, which will be finalized prior to the final database lock. Any deviations from the plan will be described and justified in the final report.

# Source Documents and Access to Source Data/Documents

Each participating site will maintain appropriate medical and research records for this trial, in compliance with ICH E6 GCP, Section 4.9 and regulatory and institutional requirements for the protection of confidentiality of subjects. Site monitoring is conducted to ensure the human subject protection, study procedures, laboratory, study intervention administration, and data collection processes are of high quality and meet sponsor, GCP/ICH and, when appropriate, regulatory guidelines. Site visits may be conducted by an authorized representative of QIMR or other regulatory agencies to inspect study data, subjects’ medical records, and CRFs in accordance with ICH guidelines, GCP and the respective local and national government regulations and guidelines.

The CI will permit authorized representatives of QIMR and the respective local and national health authorities to inspect facilities and records relevant to this study if needed.

According to ICH GCP, source data correspond to all information in original records and certified copies of original records of clinical findings, observations or other activities in clinical trial necessary for the reconstruction and evaluation of the trial. Source data are contained in source documents and these correspond to ALL original documents, data and records in either hard or electronic format. The source documents include, but not limited to, the hospital records, clinical and office charts, laboratory findings, notes, memoranda, participants diaries or evaluation checklists, pharmacy dispensing records, recorded data from automated instruments, copies or transcriptions certified after verification as being accurate and complete, participant files and records kept in the pharmacy, at the laboratories and other departments involved in the clinical study.

The data will be collected and recorded in the medical record or the source documents first by the CI or the designated clinical personnel and then entered onto the case report form (CRF).

The CI and the clinical personnel involved in the clinical study will ensure that all recorded data are accurate, complete and consistent as in accordance with the ICH GCP requirements and the site specific protocol for completion of documentation. The clinical staff involved in the study will also ensure that all records for receipt, shipment and other disposition of the study products are maintained, complete and accurate during the clinical study.

The CI is responsible to ensure that all data reported on the CRF and derived from the source documents are consistent and any discrepancies explained. To protect the confidentiality of the participants, the accuracy and the completeness of the study records only the CI and the study personnel designated by the CI will be granted an access and authorization to enter additional data to the source data. The CI will ensure that the designated study personnel are qualified appropriately and familiar with the ICH GCP requirements for conductance of clinical trial.

Upon request, the CI will permit direct access to source data/documents for trial related monitoring, audit, HREC review and regulatory inspection(s) by the sponsor or their appropriately qualified delegate and inspections by TGA. Direct access includes examination, analysis, verification and reproduction of records and reports that are important to the evaluation of the trial.

Forms for use as source documents will be derived from the CRFs.

# Quality Control and Quality Assurance

The study will be conducted in accordance to the written clinical site standard operating procedures that reflect the quality assurance (QA) and the quality control (QC) systems implemented in this study. The CI will ensure that the quality control procedures for collection, management and analysis of the data are implemented and followed in this study, as described in the clinical site specific Quality Management SOP that includes the process and the frequency of the QA audits, QC reviews, documentation involved and audit tools used.

The monitors appointed by the sponsor or inspectors will verify during monitoring visits, inspections or audits whether the implemented QA/QC system ensures that the study is being conducted and data generated, documented (recorded) and reported in compliance with this protocol, ICH GCP guidance, and the TGA requirements.

The clinical site will provide direct access to all trial related sites, source data/documents and CRFs for the purpose of monitoring and auditing by the QIMR or QIMR’s designee or inspections by TGA inspectors.

# Ethics/Protection of Human Subjects

This study will be conducted according to the principles of respect for persons, beneficence (including minimization of harms and maximization of benefits), and justice as stated in the Belmont Report and in compliance with the provisions for the protection of the rights and welfare of human research subjects set forth in World Medical Association Declaration of Helsinki – Ethical Principles for Medical Research Involving Human Subjects (2008), NH&MRC National Statement on Ethical Conduct in Human Research Humans (2007), Notes for Guidance on Good Clinical Practice – Annotated with TGA Comments (CPMP/ICH/135/95), as adopted by the Australian Therapeutic Goods Administration (July 2000) and current ethics approved Clinical Trial Protocol.

## Ethical Standard

The investigators involved in this study will ensure that this study will be fully conducted according to the current version of the Declaration of Helsinki, the International Conference for Harmonization Good Clinical Practice (ICH-GCP) regulations [3], CIOMS [33], International Ethical Guidelines of Biomedical Research Involving Human Subjects Participants (2002) and the National Statement on Ethical Conduct in Research Involving Humans [2], whichever affords the greater protection to the human subjects.

## Institutional Review Board/Ethics Committee

The investigators will ensure that all relevant documentation, including the study protocol, investigators brochure, the informed consent and all advertising documents are submitted to QIMR-HREC.

The study will not commence until approval is obtained from QIMR-HREC and it has been confirmed that TGA is notified. If the protocol is amended to significantly affect the safety of the participants in this study, such as information that has impact on the risks, clinical decision making, procedures or enrollment of participants in the study, the sponsor or designee must submit the amended protocol to QIMR-HREC for a review and pre-approval prior to implementing any of the proposed amendments in this study. The TGA sponsor or designee must ensure that TGA is informed of any QIMR-HREC approved protocol amendments.

However, if the protocol is revised only to include administrative amendments, the changes will not require pre-approval from QIMR-HREC. Submission of these changes to QIMR-HREC will not occur until the annual reporting timeframe.

## Informed Consent Process

Informed consent is required from all participants in the study. All consent forms will be approved by the QIMR-HERC prior to their use in the informed consent process. The process of obtaining and documenting informed consent of the study participants will be carried out in accordance with the guidelines for ICH GCP [4] and NHMRC National Statement on Ethical Conduct in research Involving Humans (2007) [2]. The informed consent process will be initiated by the investigators or a qualified study person designated by the investigators. The process will involve assurance that the participants have capability to make decision by themselves on whether to participate voluntarily in this study or not. It will also involve an assessment of whether the participants have an understanding of the study and the events/expectations involved in the study.

Once it is confirmed that the participants understand the study and are capable to make decisions by themselves, all participants will be asked to sign the consent form to indicate the participant’s agreement to be screening for eligibility to participate in the study..

If during the screening process, the laboratory test(s) are found clinically abnormal the participants will be referred to appropriate counseling and/or medical services.

## Exclusion of Women, Minorities, and Children (Special Populations)

Registration of pregnant women and persons younger than 18 years in this study will be restricted, because no sufficient safety data are available for the study product and their effects on the developing immune system in children or their effects on the unborn baby.

## Participant Confidentiality

The study protocol, documentation and all collected data including laboratory testing and results will be held in strict confidence and their access will be limited only to authorized personnel, including the investigators, the clinical personnel involved in this study, the sponsors and their agents. No information concerning the study or the data will be released to any unauthorized third party without prior written approval of QIMR.

Inspection of the documents and records maintained by the investigators such as medical records (office, clinical or hospital) and pharmacy records for all participants participating in the study may be available to the study monitor, a TGA inspector or other authorized personnel by the QIMR, HREC or designee.

## Study Discontinuation

The Sponsor, Principal Investigator(s), Ethics Committee (EC) and Regulatory Authorities independently reserve the right to discontinue the study at any time for safety or other reasons. This will be done in consultation with the Sponsor where practical. In the occurrence of premature trial termination or suspension, the above mentioned parties will be notified in writing by the terminator/suspender stating the reasons for early termination or suspension (with the exception of the sponsor’s responsibility for notifying the Regulatory Authorities). After such a decision, the Sponsor and the Investigator will ensure that adequate consideration is given to the protection of the subjects’ interest. The investigator must review all participating subjects as soon as practical and complete all required records.

In the event of discontinuation of the study, the study will be unblinded first and all participating participants will be asked to complete an end-of-study evaluation (section 7.6). If the study is discontinued, no further participants will be dosed.

## Subject Compensation

Volunteers who complete the study will be paid $1565 compensation for their participation. Volunteers who withdraw or are withdrawn from the study will be compensated on a fractional basis for their involvement unless they are withdrawn as a consequence of their misconduct. Reserve volunteers who do not participate in the study will be paid $150 compensation for the inconvenience associated with their attendance for screening and for their attendance on the dosing day of their respective cohort, in case they are required to participate.

## Future Use of Stored Specimens

Consent must be obtained from the all participants in this study to maintain their specimens for further use. The consent form will be submitted to QIMR-HREC for approval in accordance to the relevant QIMR SOPs prior to its use in the consent process. All samples will be stored at QIMR in accordance to the laboratory SOPs. The investigators will ensure that the confidentiality will be maintained continuously in all further studies that involve use of these specimens. The vials containing the specimens of the consented participants will be letter coded and the identifying information will not be released to any unauthorized third party. No genetic testing will be performed on these specimens.

There are no benefits to participants in the collection, storage and subsequent research use of specimens. Reports about future research done with participant samples will NOT be kept in their health records, but participant’s samples may be kept with the study records or in other secure areas. Participants can decide if they want their samples to be used for future research or have their samples destroyed at the end of the study. A participant’s decision can be changed at any time prior to the end of the study by notifying the study doctors or nurses in writing. However, if a participant consents to future use and some of their blood has already been used for research purposes, the information from that research may still be used.

## Liability/indemnity/insurance

The study sponsor will ensure sufficient insurance is available to enable it to indemnify and hold the investigator(s) and relevant staff as well as any hospital, institution, ethics committee or the like, harmless from any claims for damages for unexpected injuries, including death, that may be caused by the subject’s participation in the study but only to the extent that the claim is not caused by the fault or negligence of the volunteers or investigator(s). The sponsor adheres to the guidelines of Medicines Australia for injury resulting from participation in a company-sponsored trial, including the provision of “No-fault clinical trial insurance”.

# Data Handling and Record Keeping

The investigator is responsible to ensure the accuracy, completeness, legibility and timeliness of the data reported.

**Source Documents and Electronic Case Report Forms**

The investigators will ensure that the confidentiality of all clinical data is maintained continuously during the clinical study as required by the ICH GCP guidelines [3]. Participants will be assigned with a unique identifier such as numbers or codes at the commencing of the clinical study. The entry of data, as well as the access to the medical files or the source documents and CRFs will be also restricted to CI and the clinical study personnel designated by the CI.

Upon request, the CI will permit direct access to source data/documents for trial related monitoring, audit, QIMR-HREC review and inspection(s) by the sponsor or its designee and TGA inspectors.

Copies of CRFs will be uses as source documents and will be maintained for recording data for each participant enrolled in the study. All source documents should be completed in a neat, legible manner to ensure accurate interpretation of data. Black or blue ink is required to ensure clarity of reproduced copies. When making changes or corrections, cross out the original entry with a single line and initial and date the change. DO NOT ERASE, OVERWRITE, OR USE CORRECTION FLUID OR TAPE ON THE ORIGINAL.

## Participant Confidentiality

Participant confidentiality is strictly held in trust by the participating investigators, their staff, and the sponsor(s) and their agents. This confidentiality is extended to cover testing of biological samples, in addition to the clinical information relating to participating subjects.

The study protocol, documentation, data and all other information generated will be held in strict confidence. No information concerning the study or the data will be released to any unauthorized third party, without prior written approval of the sponsor.

The study monitor or other authorized representatives of the sponsor may inspect all documents and records required to be maintained by the Investigator, including but not limited to, medical records (office, clinic or hospital) and pharmacy records for the participants in this study. The clinical study site will permit access to such records.

## Data Management Responsibilities

Data handling and record keeping will be restricted to the investigators and the designated clinical personnel involved in the clinical study. The clinical investigator and the designated clinical personnel involved in the study are responsible to review the source data and to ensure that the data recorded are complete and accurate, the AE and SAE are reported in compliance with this protocol, ICH GCP guidelines and the TGA requirements.

Collection of the data is the responsibility of the designated clinical personnel involved in this study under the supervision of the clinical investigator. During the study, the clinical investigator or designee is required to maintain complete and accurate documentation for the clinical study.

Q-Pharm will be responsible for data management and quality review. The CI will be responsible for analysis, and reporting of the study data.

## Data Capture Methods

Clinical data (including AEs, concomitant medications, and reactogenicity data) will be recorded by designated Q-Pharm study personnel. Clinical data will be entered directly from the source documents.

## Types of Data

Types of data that will be collected in this study include:

• Safety data (solicited local and systemic responses, unsolicited adverse events and Serious Adverse events, changes in the blood and/or urine parameters, ECHO/ECGs)

• Laboratory data (clinical, immunological)

• Outcome measure data (reactogenicity, level of specific IgG, neutralization effect of the produced peptide specific IgGs)

## Timing/Reports

Reports regarding the collected safety data in the study will be ongoing. Clinical and AEs will be sent by the CI to SMC for a review as indicated in section 4 of this protocol.

In accordance to the regulatory and sponsor’s requirements, the CI will ensure that the sponsor for TGA QIMR is kept informed regarding the status of the clinical study in a timeline as specified in this protocol. The minimum reporting requirements in the conduct of the clinical study are:

• SAE report (please refer to section 9.3)

• Protocol Deviation Reports (Section 15.6)

• Clinical Study Report (at completion/early termination of the clinical study)

• Annual reports required for ongoing ethics approval and regulatory compliance

The CI or designee will ensure that the Investigator Site File (ISF), in which all study related reports and the clinical trial essential documents are filed, is maintained and current as per requirements.

## Study Records Retention

The investigators will ensure that all documents related to the clinical study are retained for a minimum of 15 years following the completion of the study as per QIMR sponsor requirements. Written agreement from QIMR must precede destruction of the same which will not be until at least 2 years after the formal discontinuation of clinical development of the investigational product and the regulatory authority TGA is notified. All records and reports for the investigational product will also be retained for at least 2 years after the shipment and delivery of the vaccine is discontinued and the regulatory authorities have been notified.

## Protocol Deviations

Protocol deviation is any incident involving non-compliance with the written clinical trial protocol approved by the QIMR-HREC in accordance to the Good Clinical Practice (GCP) guidelines. The non compliance may either be part of the study participant, the investigators or the clinical site personnel.

It is the responsibility of the site to use continuous vigilance to identify and report deviations within 5 working days of identification of the protocol deviation, or within 5 working days of the scheduled protocol-required activity.

It is the responsibility of the clinical staff involved in the study to identify and report any deviations from the protocol in accordance with site specific standard operating procedures/work instructions. The CI is responsible to ensure the implementation of the committee’s requirements following the review of the protocol deviations.

All deviations must be reported promptly to QIMR-HREC via e-forms system.

Any deviations from the protocol must be recorded in the study participant source documents. A copy of the QIMR Protocol Deviation Form must be maintained in the regulatory file and the CTMF (QIMR).

# Publication Policy

Publication and reporting of results and outcomes of this trial will be accurate and honest, undertaken with integrity and transparency and in accordance with QIMR’s Publication Policy. Publication of results will be subjected to fair peer-review. Authorship will be given to all persons providing significant input into the conception, design, execution or reporting of the research according to the QIMR Statement of Record Integrity. No person who is an author, consistent with this definition, will be excluded as an author without their permission in writing. Authorship will be discussed between investigators prior to study commencement (or as soon as possible thereafter) and reviewed whenever there are changes in participation. All conflicts arising through disputes about authorship will be reviewed by the QIMR Director. Acknowledgment will be given to collaborating institutions and hospitals and other individuals and organizations providing finance or facilities. Participant confidentiality will be maintained by referring to individual participants by their identifying code used in the trial. Data will not be released publicly until the manuscript is accepted for publication. In the case of no publication, information will be released only to the public and media in accordance with QIMR’s Corporate Media Strategy Policy.

It is a responsibility of QIMR to register this study in an acceptable public trial registry such as The **Australian New Zealand Clinical Trials Registry (**ANZCTR) which is sponsored by the NHMRC. This study must be registered on before participant enrollment.

# Literature References

[1] World Medical Association. World Medical Association Declaration of Helsinki – Ethical Principles for Medical Research Involving Human Subjects 2008.

[2] National Health and Medical Research Council (NHMRC). National Statement on Ethical Conduct in Research Involving Humans. 2007.

[3] The International Conference on Harmonization Good Clinical Practice E6 (ICH-GCP).

[4] Therapeutic Goods Administration (TGA). Note for Guidance on Good Clinical Practice (CPMP/ICH/135/95). 2000.

[5] World Health Organization. World Health Report. Geneva: Office of Publications, World Health Organization; 2000 2000.

[6] Carapetis J. A review of WHO activities in, the burden of, and the evidence for strategies to control group A streptococcal diseases: Part 3 - The current evidence for the burden of group A streptococcal diseases. Melbourne: University of Melbourne; 2004 2 March 2004.

[7] Carapetis JR, Steer AC, Mulholland EK, Weber M. The global burden of group A streptococcal diseases. Lancet Infect Dis 2005;5(11):685-94.

[8] Carapetis JR, Wolff DR, Currie BJ. Acute rheumatic fever and rheumatic heart disease in the top end of Australia's Northern Territory. Med J Aust 1996;164(3):146-9.

[9] Brandt ER, Good MF. Vaccine strategies to prevent rheumatic fever. Immunol Res 1999;19(1):89-103.

[10] Kotloff KL, Corretti M, Palmer K, Campbell JD, Reddish MA, Hu MC, et al. Safety and immunogenicity of a recombinant multivalent group a streptococcal vaccine in healthy adults: phase 1 trial. Jama 2004;292(6):709-15.

[11] Hu MC, Walls MA, Stroop SD, Reddish MA, Beall B, Dale JB. Immunogenicity of a 26-valent group A streptococcal vaccine. Infect Immun 2002;70(4):2171-7.

[12] Dale JB, Penfound T, Chiang EY, Long V, Shulman ST, Beall B. Multivalent group A streptococcal vaccine elicits bactericidal antibodies against variant M subtypes. Clin Diagn Lab Immunol 2005;12(7):833-6.

[13] McNeil SA, Halperin SA, Langley JM, Smith B, Warren A, Sharratt GP, et al. Safety and immunogenicity of 26-valent group a streptococcus vaccine in healthy adult volunteers. Clin Infect Dis 2005;41(8):1114-22.

[14] Thomson D, Thomson R. The role of the Streptococci in Scarlet Fever. Annals of the Pickett-Thomson Research Laboratory 1930;4:244-52.

[15] Massell BF. Rheumatic fever and Streptococal Infection. Boston: Harvard University Press, 1997.

[16] Beachey EH, Stollerman GH, Johnson RH, Ofek I, Bisno AL. Human immune response to immunization with a structurally defined polypeptide fragment of streptococcal M protein. J Exp Med 1979;150(4):862-77.

[17] Fox EN, Wittner MK, Dorfman A. Antigenicity of the M proteins of group A hemolytic streptococci. 3. Antibody responses and cutaneous hypersensitivity in humans. J Exp Med 1966;124(6):1135-51.

[18] Massell BF, Michael JG, Amezcua J, Siner M. Secondary and apparent primary antibody responses after group A streptococcal vaccination of 21 children. Appl Microbiol 1968;16(3):509-18.

[19] Fox EN, Pachman LM, Wittner MK, Dorfman A. Primary immunization of infants and children with group A streptococcal M protein. J Infect Dis 1969;120(5):598-604.

[20] Massell BF, Honikman LH, Amezcua J. Rheumatic fever following streptococcal vaccination. Report of three cases. Jama 1969;207(6):1115-9.

[21] Fox EN. M proteins of group A streptococci. Bacteriol Rev 1974;38(1):57-86.

[22] Cunningham MW. Pathogenesis of group A streptococcal infections. Clin Microbiol Rev 2000;13(3):470-511.

[23] Pruksakorn S, Currie B, Brandt E, Martin D, Galbraith A, Phornphutkul C, et al. Towards a Vaccine for Rheumatic-Fever - Identification of a Conserved Target Epitope on M-Protein of Group-a Streptococci. Lancet 1994;344(8923):639-42.

[24] Pruksakorn S, Galbraith A, Houghten RA, Good MF. Conserved T and B cell epitopes on the M protein of group A streptococci. Induction of bactericidal antibodies. J Immunol 1992;149(8):2729-35.

[25] Brandt ER, Hayman WA, Currie B, Carapetis J, Wood Y, Jackson DC, et al. Opsonic human antibodies from an endemic population specific for a conserved epitope on the M protein of group A streptococci. Immunology 1996;89(3):331-7.

[26] Brandt ER, Hayman WA, Currie B, Pruksakorn S, Good MF. Human antibodies to the conserved region of the M protein: opsonization of heterologous strains of group A streptococci. Vaccine 1997;15(16):1805-12.

[27] Pruksakorn S, Currie B, Brandt E, Phornphutkul C, Hunsakunachai S, Manmontri A, et al. Identification of T cell autoepitopes that cross-react with the C-terminal segment of the M protein of group A streptococci. Int Immunol 1994;6(8):1235-44.

[28] Relf WA, Cooper J, Brandt ER, Hayman WA, Anders RF, Pruksakorn S, et al. Mapping a conserved conformational epitope from the M protein of group A streptococci. Pept Res 1996;9(1):12-20.

[29] Hayman WA, Brandt ER, Relf WA, Cooper J, Saul A, Good MF. Mapping the minimal murine T cell and B cell epitopes within a peptide vaccine candidate from the conserved region of the M protein of group A streptococcus. Int Immunol 1997;9(11):1723-33.

[30] Batzloff MR, Hayman WA, Davies MR, Zeng M, Pruksakorn S, Brandt ER, et al. Protection against group A streptococcus by immunization with J8-diphtheria toxoid: contribution of J8- and diphtheria toxoid-specific antibodies to protection. J Infect Dis 2003;187(10):1598-608.

[31] Pittman PR. Aluminum-containing vaccine associated adverse events: role of route of administration and gender. Vaccine 2002;20 Suppl 3:S48-50.

[32] Mark A, Carlsson RM, Granstrom M. Subcutaneous versus intramuscular injection for booster DT vaccination of adolescents. Vaccine 1999;17(15-16):2067-72.

[33] Council for International Organizations of Medical Sciences (CIOMS).

[34] De Angelis C, Drazen JM, Frizelle FA, Haug C, Hoey J, Horton R, et al. Clinical trial registration: a statement from the International Committee of Medical Journal Editors. N Engl J Med 2004;351(12):1250-1.

[35] Working group on standardization of epidemiologic protocols for surveillance of diseases caused by *Streptococcus pyogenes*, working group 2: Acute rheumatic fever, rheumatic heart disease and acute post-streptococcal glomerulonephritis, Ver. 1.0, Jan, 2006

[36] Gaziano, T.A., Young, C.R., Fitzmaurice, G., Atwood, S. &Gaziano, J.M. Laboratory-based versus non-laboratory-based method for assessment of cardiovascular disease risk: the NHANES I Follow-up Study cohort. *Lancet* **371**, 923-931 (2008).

[37] Ashbaugh, C. D., T. J. Moser, et al. (2000). "Bacterial determinants of persistent throat colonization and the associated immune response in a primate model of human group A streptococcal pharyngeal infection." Cell Microbiol 2(4): 283-292.

[38]Garcia, A., K. Paul, et al. (2006). "Toxic shock due to Streptococcus pyogenes in a rhesus monkey (Macaca mulatta)." J Am Assoc Lab Anim Sci 45(5): 79-82.

[39]Gozalo, A., G. E. Dagle, et al. (1992). "Spontaneous cardiomyopathy resembling acute rheumatic heart disease in an owl monkey." J Med Primatol 21(7-8): 381-383.

[40]Krishna, G. and R. Iyer (1999). Animal models of rheumatic fever and rheumatic carditis. Rheumatic Fever. N. J, V. R, R. K and T. R. Washington DC, American Registry of Pathology: 195-208.

[41]Mohan, C., N. K. Ganguly, et al. (1987). "Experimental production of cardiac injury in rhesus monkeys by L-forms of group-A streptococci." Indian J Med Res 86: 361-371.

[42]Olsen, R. J., M. Ashraf, et al. (2010). "Lower respiratory tract infection in cynomolgus macaques (Macaca fascicularis) infected with group A Streptococcus." Microbial Pathogenesis 49(6): 336-347.

[43] Lucherini T, Porzio F. [Research on the induction of experimental myo-endocarditis in the rhesus monkey]. Policlinico Med. 1967 Jun;74(3):137-52
