# Supplementary material for: Evaluation of safety and immunogenicity of a group A streptococcus vaccine candidate (MJ8VAX) in a randomized clinical trial
Source: PLoS One. 2018 Jul 2;13(7):e0198658. doi: 10.1371/journal.pone.0198658 (PMC6028081; doi:10.1371/journal.pone.0198658)
Supplement: S1 Echocardiographic Exclusion Criteria — (DOCX) [file pone.0198658.s002.docx]

**S1 Echocardiographic exclusion criteria**

***Cardiac Chambers***

- Left ventricular dilatation (Based on LV diameter >29mm/m2 indexed to body surface area).
- Left ventricular systolic dysfunction (Left ventricular ejection fraction < 50%).
- Left ventricular wall thickness >11mm.
- Right ventricular dysfunction or dilatation (subjective assessment).

***Cardiac Valves / Haemodynamic Findings***

- Greater than mild valvular regurgitation (Defined as let length on colour flow imaging of >10mm, persistent through >100ms of systole on spectral Doppler and visible in at least two planes of imaging).
- Any degree of valvular stenosis, or left ventricular outflow tract obstruction.
- Pulmonary hypertension (defined as an estimated right ventricular systolic pressure of >40mmHg, calculated using the peak tricuspid regurgitant jet velocity method).

***Pericardium***

- Greater than trivial pericardial fluid (trivial defined as <5mm and not circumfrential).

***Other***

- Pre-existing significant structural valve disease (for example, but not limited to bicuspid aortic valve regardless of haemodynamic effect, mitral valve prolapse regardless of severity of regurgitation and pulmonary stenosis).
- Other significant congenital lesions (for example, but not limited to aortic coarctation, septal defect, but excluding patent foramen ovale*).

*Findings considered normal developmental variation, specifically including patent foramen ovale and prominent Eustachian valve *were not* be considered exclusion criteria
