# Supplementary material for: Evaluation of safety and immunogenicity of a group A streptococcus vaccine candidate (MJ8VAX) in a randomized clinical trial
Source: PLoS One. 2018 Jul 2;13(7):e0198658. doi: 10.1371/journal.pone.0198658 (PMC6028081; doi:10.1371/journal.pone.0198658)
Supplement: S2 Table — (DOCX) [file pone.0198658.s005.docx]

**S2 Table. J8 IgG and DT IgG Serum Concentration (µg/mL)**

|  |  | **N** | **Day 0** | **Day 28** | **Day 180^+^** | **Day 266‡** | **Day 350‡** | **Change**  **Day 0 - Day 28** |
| --- | --- | --- | --- | --- | --- | --- | --- | --- |
| **DT IgG** | **Placebo** | 2 | 35.1  (6.5-63.7) | 45.6  (-168.8-260.1) | 63.9  (-106.1-234.0) | 41.3  (-246.7-329.3) | 31.3  (-193.6- 256.3) | 10.51  (-190.3-211.4) |
|  | **Active** | 8 | 21.3  (7.0-35.6) | 247.1  (139.9-354.3) | 182.7  (91.8-273.6) | 196.7  (-38.4- 431.9) | 162.2  (-21.4-345.9) | 225.8  (125.4-326.2) |
|  | **Between SD** | 8 | 30.4 | 227.8 | 176.2 | 298.3 | 233.0 | 213.3 |
|  | **Within SD** | 20 | 18.1 | 43.9 | 11.0 | 12.5 | 9.6 | 44.6 |
|  | **P-value** |  | 0.13 | 0.089 | 0.19 | 0.40 | 0.36 | 0.058 |
| **J8 IgG** | **Placebo** | 2 | 1.31  (0.83-1.78) | 1.39  (-2.32-5.1) | 1.42  (0.36-2.49) | 0.77  (0.32-1.23) | 0.77  (0.44-1.09) | 0.09  (-3.75-3.92) |
|  | **Active** | 8 | 1.62  (1.38-1.86) | 4.54  (2.69-6.4) | 2.02  (1.45-2.59) | 1.08  (0.71- 1.45) | 1.01  (0.74-1.28) | 2.92  (1.00-4.84) |
|  | **Between SD** | 8 | 0.51 | 3.94 | 1.10 | 0.47 | 0.34 | 4.08 |
|  | **Within SD** | 20 | 0.10 | 0.24 | 0.09 | 0.05 | 0.08 | 0.26 |
|  | **P-value** |  | 0.21 | 0.12 | 0.28 | 0.30 | 0.27 | 0.17 |

+ Analyses performed with n=7 in the Active group

‡Analyses performed with n=3 in the Active group.
